# A high-resolution view of RNA endonuclease cleavage in *Bacillus subtilis*

**DOI:** 10.1093/nar/gkaf030

**Published:** 2025-01-30

**Authors:** James C Taggart, Kathryn Julia Dierksheide, Hannah J LeBlanc, Jean-Benoît Lalanne, Sylvain Durand, Frédérique Braun, Ciarán Condon, Gene-Wei Li

**Affiliations:** Department of Biology, Massachusetts Institute of Technology, Cambridge, MA 02142, USA; Department of Biology, Massachusetts Institute of Technology, Cambridge, MA 02142, USA; Department of Biology, Massachusetts Institute of Technology, Cambridge, MA 02142, USA; Department of Biology, Massachusetts Institute of Technology, Cambridge, MA 02142, USA; Department of Physics, Massachusetts Institute of Technology, Cambridge, MA 02142, USA; Expression Génétique Microbienne (EGM), CNRS, Université Paris Cité, Institut de Biologie Physico-Chimique, 13 rue Pierre et Marie Curie, 75005 Paris, France; Expression Génétique Microbienne (EGM), CNRS, Université Paris Cité, Institut de Biologie Physico-Chimique, 13 rue Pierre et Marie Curie, 75005 Paris, France; Expression Génétique Microbienne (EGM), CNRS, Université Paris Cité, Institut de Biologie Physico-Chimique, 13 rue Pierre et Marie Curie, 75005 Paris, France; Department of Biology, Massachusetts Institute of Technology, Cambridge, MA 02142, USA; Howard Hughes Medical Institute, Cambridge, MA 02142, USA

## Abstract

RNA endonucleases are the rate-limiting initiator of decay for many bacterial mRNAs. However, the positions of cleavage and their sequence determinants remain elusive even for the well-studied *Bacillus subtilis*. Here we present two complementary approaches—transcriptome-wide mapping of endoribonucleolytic activity and deep mutational scanning of RNA cleavage sites—that reveal distinct rules governing the specificity among *B. subtilis* endoribonucleases. Detection of RNA terminal nucleotides in both 5′- and 3′-exonuclease-deficient cells revealed >10^3^ putative endonucleolytic cleavage sites with single-nucleotide resolution. We found a surprisingly weak consensus for RNase Y targets, a contrastingly strong primary sequence motif for EndoA targets, and long-range intramolecular secondary structures for RNase III targets. Deep mutational analysis of RNase Y cleavage sites showed that the specificity is governed by many disjointed sequence features. Our results highlight the delocalized nature of mRNA stability determinants and provide a strategy for elucidating endoribonuclease specificity *in vivo*.

## Graphical Abstract

**Graphical Abstract:**
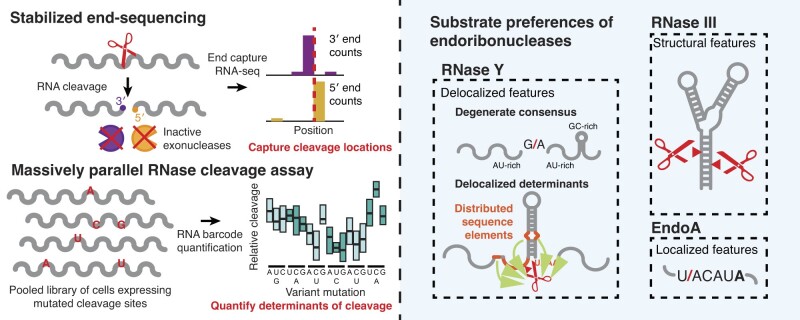

## Introduction

Predicting protein abundance from gene sequence remains an aspirational goal for quantitative biology. Decades of research has explored the encoding of rates of transcription and translation initiation [1], informing predictive models for how RNA polymerase and the ribosome recognize promoters and ribosome binding sites [2–5]. Comparatively poorly characterized, however, is post-transcriptional control of RNA abundance by ribonucleases, factors that exert widespread quantitative control on bacterial gene expression levels [6–8]. Understanding the encoding of ribonuclease cleavage sites in bacterial mRNA sequences is therefore a critical question in quantitative biology.

In the Gram-positive bacterium *Bacillus subtilis*, cleavage by an endoribonuclease is thought to initiate the decay of a large fraction of cellular mRNAs [9–12]. The cleaved RNA fragments typically have a 5′-phosphate and a 3′-hydroxyl group, which are subsequently degraded by a 5′-3′ exoribonuclease (RNase J1) and by a combination of 3′-5′ exoribonucleases (PNPase, RNase R, RNase PH, and YhaM) [13], respectively. The endoribonuclease RNase Y appears to drive the turnover of most mRNAs in *B. subtilis*, and its depletion results in the accumulation of hundreds of transcripts [9,10,12]. RNase Y cleavage is specific to certain positions and drives maturation of several well-studied operons [14–17]. Unfortunately, despite its central role in RNA metabolism, our understanding of which mRNA sequences *B. subtilis* RNase Y cleaves remains limited. Studies suggest *B. subtilis* RNase Y recognizes single-stranded RNA, is stimulated by downstream secondary structure, and prefers cleaving adenosine and uridine-rich sequence [9,14]. Orthologs of RNase Y in *Staphylococcus aureus* and *Streptococcus pyogenes*, organisms for which RNase Y is not essential and appears to play a more limited role compared to *B. subtilis*, additionally demonstrate a preference for cleavage downstream of a guanosine [7,18]. However, these limited sequence preferences cannot explain the highly specific locations of cleavage that have been observed for the *B. subtilis* RNase Y [15].

In addition to RNase Y, several other endoribonucleases contribute to mRNA decay. RNase III is thought to cleave and initiate decay for dozens of mRNAs in *B. subtilis* [19]. Although the *Escherichia coli* RNase III strictly cleaves double-stranded RNAs, the *B. subtilis* RNase III has been implicated to deviate from this rule in a way that remains unknown [19]. Other minor endoribonucleases, such as the RNase toxin EndoA (a homolog of *E. coli* MazF) and the newly discovered YloC, may also initiate mRNA decay, generating a variety of 5′ and 3′ moieties [20–23]. Their positions of cleavage and subsequent decay processes remain poorly described.

A number of studies have mapped the target repertoire of major bacterial endoribonucleases [7,19,24–27]. These measurements detect the terminal nucleotides of RNA decay intermediates and analyze their abundance upon endoribonuclease inactivation. The results are confounded by several factors. First, because cleaved decay intermediates are typically unstable, the detected end signals are often close to background levels and may be biased by differential stability. Second, the sequential actions of several endoribonucleases and/or exoribonucleases on the same mRNA may result in RNA ends that are shifted from the initial position that is cleaved by the endoribonuclease under investigation. As a result, low-throughput approaches are often required to precisely determine exact positions of cleavage for each target [14,19,28,29].

In this work, we first present a global approach to map bacterial endoribonuclease cleavage sites by combining high-resolution RNA end-mapping with genetic perturbation of all known exonucleases participating in *B. subtilis* mRNA decay. By stabilizing mRNA decay intermediates in exonuclease deficient *B. subtilis* cells, this “stabilized end-sequencing” approach revealed thousands of putative positions of endoribonuclease cleavage and a previously unknown nuclease activity targeting RNA 5′ ends. Furthermore, using a panel of endoribonuclease knockouts, we dramatically expanded the known target repertoire for RNase Y, RNase III, and EndoA. In contrast to strong motifs observed for RNase III and EndoA cleavage, we found that RNase Y cleavage sites share a surprisingly weak consensus. To probe the sequence determinants for RNase Y cleavage, we developed a massively parallel reporter assay (MPRA) to interrogate the effects of mutation on individual cleavage sites. The residues that affect cleavage activity are scattered across a >40 nucleotide region, suggesting that the highly specific RNase Y cleavage does not follow the conventional model of protein–nucleic acid interactions mediated by compact sequence or structural motifs. Instead, the ‘cleavage code’ is specified by a collection of interactions embedded in an extended region.

## Materials and methods

### Strains and strain construction

For all Rend-seq, 5′-end sequencing, and 3′-end sequencing experiments, excluding the Rend-seq measurement of GLB186, derivatives of W168 were used (Supplementary Table S1). Unless otherwise noted, strain construction was performed using standard protocols relying on natural *B. subtilis* competence [30]. BJT070 was created through SPP1 transduction of the *ylbF::erm* allele from BJT034 into CCB396 [31]. To create BJT074, genomic DNA (gDNA) from BJT034 was first transformed into SSB1002 to generate a *ylbF* knockout in a W168 background, BJT069. SPP1 transduction from CCB434 was then used to construct an *rnjA ylbF* double knockout, BJT074. CCB390 was constructed by first transferring an inducible RNase J1 allele (first described in [32]) into W168 (strain SSB1002), followed by deletion of *rnjA* through transformation with gDNA from CCB434. To construct BJT231, gDNA from GLB230 was transformed into CCB390, supplementing with 2% xylose in the liquid and solid media throughout the transformation protocol. To construct BJT129, CCB390 was transformed with gDNA from an *rppH* knockout, strain BKE30630 [33].

A supercompetent *B. subtilis* strain BJT200, based on [34], was used for MPRA experiments unless otherwise specified. This strain was constructed from W168 *B. subtilis* strain SSB1002 through insertion of an anhydrotetracycline (aTc) inducible *comK* allele at the *ganA* locus by transformation of ScaI-linearized plasmid pJT117. For experiments in which an inducible RNase J1 was required, strain BJT201 was utilized. BJT201 is a derivative of strain CCB390, a W168 *B. subtilis* strain with xylose inducible RNase J1, with an inducible *comK* added through transformation with pJT117. These strains were used for all MPRA experiments except the *tetM-cggR* data, which were collected in a *trpC* 168 strain. All plasmids were constructed by isothermal assembly.

### Cell growth and harvesting (Rend-seq, 5′-, and 3′-end sequencing)

Except for those on our *rnjB* deletion with RNase J1 depletion (strain GLB186, see below), Rend-seq, 5′-, and 3′-end sequencing experiments were performed using 2xYT with 2% xylose or glucose for RNase J1 induction or depletion as necessary. Except when we sought to deplete RNase J1, inducible RNase J1 strains were grown in the presence of 2% xylose. For all sequencing experiments, overnight cultures in 2xYT were back-diluted to an OD_600_ of 0.0002 in 20 mL 37°C 2xYT and harvested at an OD_600_ of 0.2. RNase J1 knockout strains were vortexed prior to and following inoculation to minimize clumping. To achieve RNase J1 depletion, 10 times the required volume of overnight culture was collected, washed twice with 1 mL 37°C 2xYT, and resuspended in 1 mL 2xYT. 100 μL of this resuspension was used to inoculate the final culture. Samples were harvested by adding 7 mL of culture to 7 mL of ice-cold methanol, pelleting, flash freezing with liquid nitrogen, and transferring to −80°C.

Rend-seq for strain GLB186 was performed using LB with 1 mM isopropylthio-β-D-galactoside (IPTG). Cells were grown at 37°C to an OD_600_ of 0.3, pelleted and washed, and resuspended to OD_600_ of 0.01 without IPTG. These cells were then grown and harvested at an OD_600_ of 0.3.

### End-enriched RNA sequencing and 3′-end sequencing

Excluding our first Rend-seq experiments profiling wild-type 168, 4-exo, and Δ*rnjA* strains with and without *ylbF* knockout, cell pellets were washed with 10–15 mL ice cold 10 mM Tris pH 7.0 to remove media-derived contaminants. For Rend-seq, RNA was extracted using either an RNeasy Mini Kit (Qiagen) with on-column DNase treatment, an RNeasy Plus Mini Kit (Qiagen) or through RNAsnap extraction [35], followed by Turbo DNase treatment (ThermoFisher). All 3′-end sequencing samples were purified through RNAsnap. In the RNAsnap protocol, cells were resuspended in 500 μL extraction solution (95% Formamide, 1% BME, 0.025% SDS, 18 mM EDTA) and transferred to a 2 mL tube containing 200 μL chilled 0.1 mm diameter zirconia beads. These tubes were vortexed for 10 min using a horizontal vortex adapter and incubated at 95°C for 7 min. Samples were spun at 15 000 g, after which 400 μL was removed and diluted with 1.6 mL DEPC-treated water. This mixture was precipitated with isopropanol and resuspended in 10 mM Tris, pH 7.0. At least 30 μg of RNA was then treated for 30 min at 37°C with 10 μL Turbo DNase in a 100 μL reaction. Approximately 20 μg of purified RNA was rRNA depleted using the MICROBExpress Bacterial mRNA Enrichment Kit (ThermoFisher) and precipitated with isopropanol.

Rend-seq libraries were prepared as described previously [36]. In short, rRNA depleted RNA was fragmented for 25 s (ThermoFisher #AM8740) and fragments of sizes 15–45 nt were size selected on a 15% TBE-Urea polyacrylamide gel (ThermoFisher). RNA was dephosphorylated with T4 PNK (NEB) and 3 pmol of dephosphorylated RNA was ligated to an adenylated adaptor using truncated T4 RNA ligase 2 K277Q (Jonathan Weissman). Ligated RNA was purified by denaturing polyacrylamide gel extraction (PAGE) and reverse transcribed using SuperScript III (ThermoFisher) and primer oCJ485. This cDNA was circularized using CircLigase (Lucigen) and barcodes and Illumina adapters were added through polymerase chain reaction (PCR) using Phusion (NEB).

3′-end sequencing parallels Rend-seq, with deviation in the order of reactions [37]. Prior to fragmentation, 1.8 μg of rRNA depleted RNA was ligated to an adenylated adapter as previously described. This ligation product was fragmented and 33–63 nt RNAs were purified by PAGE. From here, the library preparation is identical to Rend-seq. Note that this protocol lacks dephosphorylation prior to 3′-end ligation, such that RNA ends with a 3′ phosphate will not be captured. Both Rend-seq and 5′-end sequencing libraries were sequenced with 50- or 75-bp single-end reads on either a HiSeq 2000 or NextSeq500.

### Multiplexed 5′-end sequencing

Pellets were washed, and RNA was extracted using the RNAsnap protocol and Turbo DNase treated as described previously. rRNA was depleted from 10 μg of purified RNA with the MICROBExpress Bacterial mRNA Enrichment Kit, per manufacturer's instructions, precipitated, and resuspended in 10 mM Tris pH 7.0. 600 ng rRNA depleted RNA was then ligated to 260 pmol of the RNA adapter oJT900 (Supplementary Table S2) for 18 h at 22°C using T4 RNA Ligase 1 (NEB, based on [38]).

Following ligation, RNA was fragmented for 2 min and fragments of either size 41–71 nt (corresponding to 15–45 nt plus a 26-nt adapter) or 60–90 nt were isolated through denaturing PAGE. The larger size window was chosen to remove dimerized adapter fragments, which were abundant in sequencing libraries prepared with the smaller size-range. Fragments were dephosphorylated, polyadenylated, and reverse transcribed following the BaM-seq protocol [39]. In short, RNA fragments are sequentially dephosphorylated with T4 PNK, polyadenylated with *E. coli* poly(A) polymerase (NEB) and reverse transcribed with SuperScript III and a barcoded reverse transcription primer. The cDNA from each sample can then be pooled and purified by denaturing PAGE. To add Illumina adapters, cDNA was amplified 5–10 cycles with primers oDP128 and oDP161 [39] using Q5 DNA polymerase (NEB) and purified by PAGE. 5′-end sequencing libraries were sequenced with 75-bp single-end reads on a NextSeq500.

### Rend-seq, 5′-, and 3′-end sequencing data processing

Adapter or poly(A) sequence was trimmed from reads using Cutadapt [40] with parameters “-a ‘CTGTAGGCACCATCAAT’ -m 15” or “-a A{15} -m 15 -u 8 –rename={id} UMI={cut_prefix}.” 5′-end sequencing reads were additionally collapsed using the 8-bp unique molecular identifier (UMI) at the start of each read using custom Python scripts, requiring identical UMI sequences and read sequences within a hamming distance of 1 to be considered duplicates. Trimmed reads were then mapped to the *S. cerevisiae* genome (SacCer3) using bowtie v1.1.2 with parameters “-v 1 -k 1 –best –un” and unmapped reads subsequently mapped to the *B. subtilis* genome (NC_000964.3) with the same parameters [41]. Mapping against yeast removes contaminating 2xYT-derived signal. Using custom Python scripts, we converted the bowtie output to wig files, attributing signal from reads with a mismatch at their first position to the adjacent nucleotide to accommodate non-templated addition during reverse transcription.

### Identification of putative endonuclease sites

To detect putative sites of endoribonucleolytic cleavage, we identified positions with adjacent 5′ and 3′-ends in our end sequencing approaches. To do so, we first normalized each dataset to the total number of millions of CDS-mapped reads. At each position, we identified peaks in read density by calculating a ratio of 5′-end signal at that position to the 90% winsorized average in a window 50-nt upstream (for 3′ ends) or downstream (for 5′ ends) of that position, excluding a 2-nt gap on either side of the peak. Adjacent positions (allowing a 1-nt gap) with a ratio greater than 7.5 were grouped for the purposes of downstream 5′-end signal quantification. At each position with >10 reads per million CDS-mapped reads (RPM) in 5′-end sequencing (or >2 RPM if noted, see ‘RNase III sequence-structure motif analysis’), we detected paired 3′ ends by calculating an equivalent ratio centered around the position one nucleotide upstream in the 3′-end sequencing of the 4-exo deletion. Additionally, in the 50-nt window downstream of each position, we calculated the local density of mapped 3′ ends of the RNase J1 deletion Rend-seq data to establish that we were considering positions only in genes expressed in this strain. Putative cleavage sites were called as positions for which the average local Rend-seq depth exceeded 0.05 RPM and both the 3′- and 5′-end sequencing signals had a ratio of 7.5 over background.

Putative cleavage sites were filtered by two criteria. First, because the sequencing methods used in this work are unable to capture short RNA fragments, we did not consider positions within 40 nt of an RNA 3′ end. To identify these ends, we called peaks in 3′-mapped RNase J1 Rend-seq data using a z-score based peak calling method. At each position, a z-score was calculated as the signal minus the average in a window of 50 nt on either side of that position (excluding a gap of 2 nt on either side), divided by the standard deviation within this window. Any position for which the calculated score exceeds 8 was flagged as an RNA 3′ end. Second, we removed sites which were either within 150 nt of a mature rRNA, tRNA, or scRNA or were >500 nt from a coding region. To count putative cleavage positions which meet these criteria across all our datasets, positions that were called in either the W168 (CCB434) or phage-cured W168 (BG880) background were considered. If two adjacent positions were called across these datasets, they were counted only a single position.

To attribute putative cleavage to specific endoribonucleases, we calculated the ratio of 5′-end sequencing signal at each position in an endonuclease knockout with RNase J1 deletion (or depletion) to the average Rend-seq signal from the same strain in a window 50-nt downstream of that position, excluding a 2-nt gap. To account for differences in sequencing depth, the 5′-end sequencing and Rend-seq signal at each position was normalized to the total CDS-mapped reads in each sample. Additionally, signal from positions previously flagged as grouped in the 5′-end sequencing were summed, with the cleavage assigned upstream of the 5′-most grouped position. This ratio of 5′-end sequencing to Rend-seq signal is calculated for the corresponding endoribonuclease-expressing RNase J1 deficient strain (e.g. a phage-cured RNase J1 deletion when considering RNase III). The ratio in the endoribonuclease expressing strain is then divided by the ratio in the endoribonuclease knockout strain to produce a ‘sensitivity’ score. This approach is similar to that used to map RNase III sites in previous work [19].

Putative cleavage positions with a sensitivity score that exceeds 10.69 for a particular endoribonuclease knockout are considered cleavage sites for that enzyme. This threshold was empirically determined by fitting of the distribution of log-transformed sensitivity values for the RNase Y knockout to the sum of two Gaussian distributions and finding the point at which 95% of this fit is contributed by the underlying Gaussian with the larger mean (see Fig. 5A). Any position for which the local Rend-seq density in the endonuclease knockout strain (as calculated above) falls below 0.05 RPM is excluded in this endonuclease assignment to avoid spuriously calling positions in genes that are downregulated in this condition. When calculating the fraction of putative cleavage sites that are dependent on a particular protein, only cleavage sites that were identified in an RNase J1 knockout of the same background were considered (e.g. only sites appearing in the phage-cured RNase J1 knockout BG880 were used when calculating the fraction of sites sensitive to RNase III knockout, see Supplementary Table S1).

### Determination of RNase III site duplex length, genomic distance, and structural distance

To identify potential intramolecular pairing between RNase III cleavage positions, we first identified all RNase III-dependent cleavage positions within 1 kb of one another. The distance between sites in the primary sequence (‘genomic distance’) is reported in Fig. 3D. We then extracted the intervening RNA sequence plus 50 nt on either side for each pair of positions and calculated its minimum-free energy (MFE) structure using RNAfold from ViennaRNA v2.4.14 with default parameters [42]. We used this MFE structure and custom Python scripts to calculate the length of the overhang generated (if any) by RNase III cleavage at this pair of sites (‘structural distance’), reported in Fig. 3E. The duplex length for each structure was manually determined, allowing ≤2 unpaired bases between base pairs.

### RNase III sequence-structure motif analysis

For analyses of sequence content within RNA duplexes cleaved by RNase III, we considered an expanded set of putative cleavage sites, using a threshold of >2 reads per million CDS-mapping reads in 5′-end sequencing (rather than the previous >10 RPM). Results using this threshold are summarized in Supplementary Tables S5 and S6. As described above, from this set of putative cleavage sites we identified pairs of sites within 1 kb of one another, calculated an MFE secondary structure, and determined the overhang length generated by cleavage, retaining only pairs which produced a 2-bp 3′ overhang.

Using the predicted MFE structures, we determined the position of each base pair relative to the two positions of cleavage. The position-wise frequency of each type of base-pairing interaction (A-U, U-A, G-C, C-G, U-G, or G-U) and fraction of sites for which that position was paired was then determined. In cases where an asymmetric bulge was observed, those bases were included as additional unpaired positions, rather than skipping over them in our indexing of base-pairs. To compare our data to those previously-published for *E. coli* [26], we calculated the difference between the frequency of each pairing type in our data and the equivalent observed at that position by Altuvia *et al.*

### Local secondary structure prediction for RNase Y sites

To determine folding propensity of sequences proximal to endoribonuclease cleavage sites, the *B. subtilis* genome was tiled with 40-mers and folded with RNAfold from the ViennaRNA v2.4.14 package with default parameters [42]. MFE values were assigned to the center position within each k-mer and summarized in wig files. Values were extracted in 150-nt windows centered around putative endoribonuclease cleavage positions and a position-wise mean was calculated. Cleavage sites within 75 nt of a 3′ or 5′-end detected in wild-type W168 Rend-seq data (identified in strain SSB1002 using our z-score based peak calling method) were excluded. To generate a background model that controls for sequence context in the −1/+1 positions, this analysis was repeated 100 times sampling an equivalent number of random positions within coding regions that are centered on GA dinucleotides (the most enriched dinucleotide flanking putative RNase Y sites), excluding called cleavage positions. The 25th and 75th percentile at each position across all predicted position-wise average folding curves is shaded in Fig. 5D.

### Information content and k-mer logo generation

To generate sequence logos, the sequences in a 30-nt window surrounding all putative cleavage sites for a given enzyme were aggregated into a FASTA file. A background set of 30-mers evenly sampled every 100 nt from *B. subtilis* coding regions (with sampling beginning after the start codon) was generated as a background set. kpLogo v1.1 [43] was then used to generate information content and k-mer logos with parameters “-bgfile {background_set} -startPos 16 -pc 0.01.”

### MPRA plasmid library construction

Plasmid libraries were constructed through restriction cloning of randomized mRNA processing site sequences into reporter plasmids pJT028 (*tetM*) or pJT124 (*aprE*). Inserts were constructed through a two-step protocol. Oligonucleotides were ordered from IDT with a 9% error rate (3% per non-native nucleotide) over the specified positions. Supplementary Table S7 contains the details of all MPRA experiments, which are each given a unique identifier (e.g. “experiment 3”). 10 fmol of oligo was PCR amplified for 12 cycles using Q5 DNA polymerase. For the MRPA experiments labeled 2a, 2b, 4a, 4b, and 6, oJT837 was amplified with oJT851/852. For the MPRA experiments labeled 8a, 8b, and 9, oJT839 was amplified with oJT857/858. For the MPRA experiments labeled 1a, 1b, 3, 5, and 7, oJT592 and oJT593 were mixed, denatured, annealed, and extended with Q5. All of these products were diluted 1:10 into a second reaction in which they were amplified and barcoded through four cycles of PCR with Q5 using oJT548/594 or oJT855/859 for all *cggR* and *glnR* inserts, respectively. Completed inserts were cleaned up with a DNA Clean & Concentrator 5 column (Zymo Research).

To construct the plasmid pool, the insert and 4 μg plasmid backbone were digested with SalI-HF and NheI-HF (NEB) for 60 min at 37°C and purified with a DNA Clean & Concentrator 5 column. 400 ng cut plasmid and insert were mixed at an approximately 1:10 ratio and ligated through a 5–15 min treatment with QuickLigase (NEB) at 25°C. This reaction was then cleaned up with a DNA Clean & Concentrator 5 column and transformed into electrocompetent *E. coli* (NEB 10-beta or NEB 5-alpha cells). Transformants were plated on LB with 100 μg/mL carbenicillin in 245 mm square bioassay dishes (Corning) and grown at 37°C overnight. Cells were then scraped off the plates for ZymoPure II Maxiprep plasmid extraction (Zymo Research).

### Transformation and integration of MPRA library into *B. subtilis* genome

To introduce our plasmid libraries into *B. subtilis*, we utilized an inducible competence system mediated by an aTc-inducible *comK* allele introduced into BJT200 and BJT201, based on [34]. A colony was picked into LB (with 2% xylose for BJT201) and allowed to grow to an OD_600_ of 1.0 at 37°C with vigorous shaking. At this point, 10 ng/mL aTc was added to induce expression of ComK. Cells were shaken at 37°C for an additional 2 h, at which point 100 μL per mL culture of plasmid pool linearized with either ScaI-HF or AfeI (NEB) was added. This restriction digest was prepared per the manufacturers protocol with 1 μg plasmid per 50 μL reaction. The cells were cultured with DNA for 1.5 h, then pelleted, resuspended in residual media, and plated on 245 mm bioassay dishes containing 100 μg/mL spectinomycin or 5 μg/mL chloramphenicol. The *cggR-tetM* library was transformed using standard protocols relying on natural competence. After growth overnight at 37°C, cells were scraped into LB with the appropriate antibiotic and approximately 500 million cells were back-diluted into 200 mL LB with antibiotic. This culture was grown for approximately 4–8 h, and 1 mL aliquots of culture were mixed 1:1 with 40% glycerol and frozen at −80°C.

### Cell growth and harvesting (MPRA)

For all MPRA experiments, cells were grown in LB medium with an inducer (20 ng/mL aTc or 2% xylose) when noted. When applicable, 2% xylose was consistently used to maintain RNase J1 expression, which was substituted for 2% glucose when RNase J1 depletion was required. To collect samples, a single cryovial of a *B. subtilis* library was thawed, washed twice with 10 mL LB, and grown overnight in 200 mL of LB at 37°C in a 2.8 L flask with vigorous shaking. These cultures were back-diluted to an OD_600_ of 0.0003 in 200 mL LB with library inducer and grown at 37°C until an OD_600_ of 0.3, at which point samples were harvested. Two cell pellets were harvested per sample: First, for RNA, 7 mL culture was added to 7 mL ice cold methanol, pelleted, and flash-frozen in liquid nitrogen. Second, for gDNA, 14 mL of culture was pelleted, and frozen in liquid nitrogen. Cell pellets were stored at −80°C until nucleic acid extraction.

### MPRA RNA and DNA sequencing

Three sequencing libraries were prepared for each experiment: one to quantify the barcodes in cellular RNA, one to quantify barcodes in the gDNA, and one to map barcodes to variant sequence. For RNA barcode quantification, RNA was extracted from cell pellets with gDNA depletion using an RNeasy Plus Mini Kit. For MPRA experiments labeled 1a, 1b, 2a, 2b, and 9 (Supplementary Table S7), 40 μg of total RNA was reverse transcribed using primer oJT442 and SuperScript III. This cDNA was then run on a 6% TBE-Urea gel, purifying 300–500 nt for full-length *cggR* and *glnR* constructs (experiments 1a, 2a, 9) and 100–250 nt for cleaved *cggR* constructs (experiments 1b and 2b). For the remaining experiments, cleaved RNA was isolated by gel purification of RNAs ranging from 200 to 400 nt for *tetM-cggR* sites, 150 to 300 nt for *aprE-cggR* sites, and 150 to 350 nt for *aprE-glnR* sites using a 6% TBE-Urea gel. This size selected RNA was reverse transcribed using primer oJT442 and SuperScript III and size selected on 6% TBE-Urea gel, extracting approximately 120–200 nt for *cggR* libraries and 100–200 for *glnR*. For all experiments, size-selected cDNA was amplified using Phusion DNA polymerase, adding a barcode and Illumina adapters with a Truseq indexing primer and an isoform-specific primer (Supplementary Table S2). These isoform-specific primers were oJT441/oJT1290 for cleaved/full-length products from *cggR* constructs or oJT890/oJT1291 for cleaved/full-length products from *glnR* constructs. The resulting libraries were sequenced with 50- or 75-bp single-end reads on an Illumina NextSeq500.

To prepare both libraries for gDNA barcode quantification and barcode to variant sequence mapping, a gDNA was extracted from a cell pellet using the Wizard Genomic DNA Purification Kit (Promega). Both gDNA-derived libraries were generated using a two-step Phusion PCR protocol. An initial 2 cycle PCR was run using 4 μg of purified nucleic acid per 50 μL PCR reaction (scaled as needed for library complexity) with primers oJT444/445 for *cggR* upstream mutations, oJT1125/445 for *cggR* downstream mutations, and oJT891/445 for *glnR* downstream mutations. The untranslated *cggR* library was instead amplified with oJT942 and oJT445. This first PCR reaction was cleaned up with a double-sided select-a-size DNA Clean & Concentrator column (Zymo) and a DNA Clean & Concentrator 5 column. A quarter of this reaction was then used for each of two different PCRs. First, for barcode quantification, amplification was performed using oJT441 or oJT890 with our Truseq indexing primer and size selected on an 8% TBE gel. These libraries were sequenced with 50- or 75-bp single-end reads on an Illumina NextSeq500. Libraries mapping barcodes to variant sequence were amplified using primers oJT446/447, size selected on an 8% TBE gel and sequenced with either 75- or 150-bp-paired end reads on an Illumina MiSeq.

### Barcode to variant sequence mapping and barcode quantification

Barcode quantification and variant sequence identification was handled using custom Python scripts. To determine the variant sequence associated with each barcode, reads from gDNA-derived libraries were directly parsed, with one read pair mate capturing the barcode and the other the variable region. Variants with ≥1 mutation in the constant region proceeding the barcode were discarded. To mitigate the effects of rare mutations or sequencing errors, variant sequence assignment required ≥3 reads per barcode. If, among these reads, the second-most frequent sequence was at least 25% as frequent as the top sequence, the barcode was discarded entirely.

Barcodes were counted by directly parsing the sequencing reads derived from both gDNA and RNA, with the barcode appearing at a fixed position within the read. As before, any variant with mutations in the constant sequence proceeding the barcode was discarded. Additionally, any two barcodes of identical sequence and UMI were counted as a single read. The number of unique reads mapping to each variant barcode was calculated for both the RNA and gDNA-derived samples, and the relative accumulation of processed mRNA for each variant was read out as ratio of RNA to gDNA-derived reads. Barcodes were then grouped by mutation, thresholded based on read count, and RNA:gDNA ratios normalized by that of the median wild-type variant.

## Results

### Stabilized end-sequencing reveals positions of endoribonucleolytic activity

**Figure 1.**
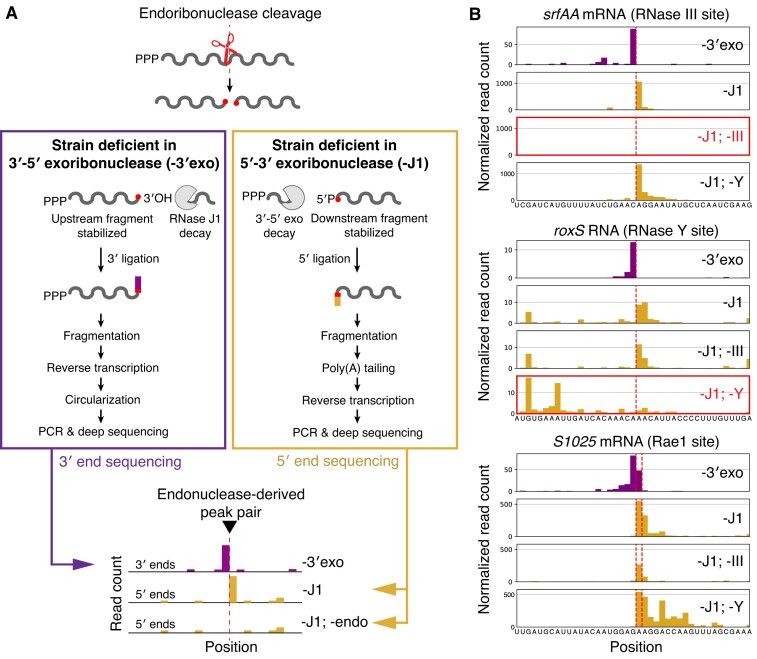
Workflow and validation of endoribonuclease cleavage mapping approach (**A**) Deletion of exoribonucleases results in stable accumulation of RNA decay intermediates. A schematic mRNA is shown with endoribonuclease cleavage (scissors) occurring, with remaining exoribonucleases indicated with Pacman symbols. RNAs are shown with 5′-P and 3′-OH characteristic of the most common endoribonucleases in *B. subtilis*. Newly generated RNA ends can be mapped through RNA ligation and sequencing. Adapters used to capture RNA ends indicated in purple for 3′-end sequencing and yellow for 5′-end sequencing, with matching color scheme in schematic data below. (**B**) Validation of endonuclease cleavage site detection using known positions of endoribonucleolytic cleavage by RNase III, RNase Y, and Rae1 [19,21,49]. Yellow indicates 5′-end sequencing and purple indicates 3′-end sequencing. Dotted vertical line represents manually annotated cleavage positions. Plotted are reads per million CDS-mapping reads, normalized to the average 3′-mapped Rend-seq RPM in this window.

Without additional nucleolytic activity, positions of endoribonuclease cleavage can be mapped by observing the termini of their cleavage products. *In vivo*, however, exonucleases typically rapidly degrade endonuclease cleavage products, masking the original RNA endonuclease cleavage sites. If exoribonucleolytic activity targeting either 3′ or 5′ ends is ablated, endoribonuclease cleavage should result in stably accumulating products whose ends correspond to positions of cleavage (Fig. 1A). Knockouts of all known exonucleases in either the 5′ or 3′ direction are viable in *B. subtilis*, enabling such a measurement [44,45]. If RNA ends are separately mapped in a knockout of RNase J1, the only known 5′-3′ exonuclease, and a knockout of all four known 3′-5′ exonucleases (termed ‘4-exo’, a knockout of *yhaM, rph, pnpA*, and *rnr*), exact positions of endoribonucleolytic activity can be captured as pairs of adjacent 3′ and 5′ ends that appear across the two strains.

Based on this principle, we implemented a ‘stabilized end-sequencing’ approach to create a transcriptome-wide map of endoribonuclease activity by capturing the 3′ hydroxyl and 5′ monophosphate groups generated by endonucleases such as RNases Y and III (the primary endonucleases targeting mRNAs in *B. subtilis*) (Fig. 1A; [21,46]). To quantitatively capture 3′ hydroxyl groups, we utilized a previously established method for RNA 3′ ligation and deep sequencing, hereafter referred to as 3′-end sequencing (Fig. 1A, left; [37,47,48]). To complement 3′-end mapping, we specifically captured 5′ monophosphates through T4 RNA Ligase 1 treatment, allowing quantification of cleavage-derived 5′ ends by deep sequencing, hereafter referred to as 5′-end sequencing (Fig. 1A, right). By performing 3′-end sequencing on a 4-exo knockout (strain CCB396) and 5′-end sequencing on an RNase J1 knockout (strain CCB434), we captured 3′/5′-end pairs derived from endoribonuclease activity transcriptome-wide. Combining these exoribonuclease perturbations with endoribonuclease knockouts, we can attribute these cleavage signatures to specific enzymes. End-enriched RNA sequencing (Rend-seq) can additionally be performed to quantify expression of putative endoribonuclease targets and provide an orthogonal RNA end-mapping approach [36].

This stabilized end-sequencing strategy robustly captures positions of previously documented endoribonucleolytic cleavage sites. Among 13 previously characterized sites cleaved by either RNase Y, RNase III, or Rae1 that were individually detected [14,16,19,21,28,29,49], we identified at least one clear 3′/5′ pair at or near the annotated cleavage position (Fig. 1B, Supplementary Fig. S1). 5′-end sequencing of RNase Y + J1 or III + J1 double mutant strains (CCB760 and BG879) confirmed that we can detect cleavage by both endoribonucleases and accurately assign the enzymes responsible for cleavage (Fig. 1B).

Importantly, the paired end-mapping strategy provides clear advantages over measurements of a single end type. Because we capture both RNA ends generated by endoribonuclease activity, adjacent positions of cleavage can be resolved, as appears to be the case for the first characterized substrate of Rae1, *S1025* (Fig. 1B, bottom). Without these two paired channels of information, it is difficult to determine whether signal at adjacent positions is derived from multiple positions of endoribonucleolytic cleavage or residual exoribonuclease activity by unknown enzymes, the latter of which may be occurring after endoribonucleolytic cleavage of the RoxS RNA (Fig. 1B, middle). Precisely resolving cleavage positions becomes particularly relevant in cases such as the endoribonucleolytic cleavages observed in *hbs*, where many nearby positions are known to be subject to RNase Y cleavage (Supplementary Fig. S1 [14]).

**Figure 2.**
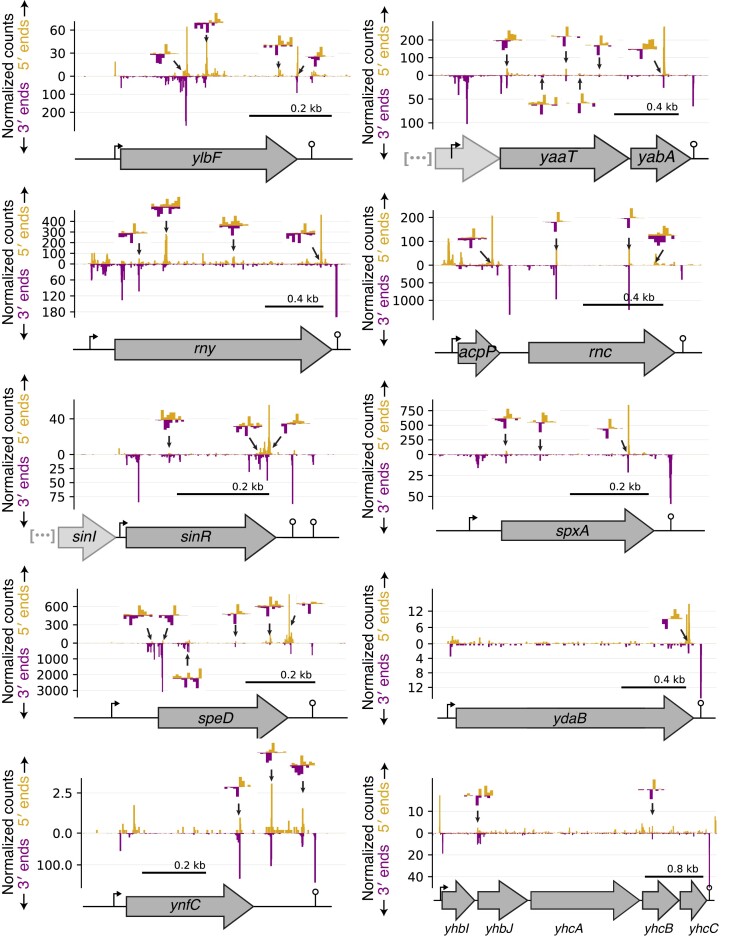
Cleavage within transcripts known to be destabilized by endoribonucleolytic cleavage. 5′- and 3′-end sequencing data are shown in yellow and purple, respectively. Plotted are reads per million CDS-mapping reads. Manually annotated putative cleavage sites (positions of adjacent 3′ and 5′ read density) are highlighted with arrows. Insets show highlighted sites at single-nucleotide resolution, with the *Y*-axis in each direction rescaled to the maximal value within the inset region. Annotated promoters and transcriptional terminators, indicated by bent arrows and lollipops, were identified using Rend-seq in SSB1002. Sequence context and RNase Y dependence of highlighted sites shown in Supplementary Fig. S2. Consistent with a recent report [50], multiple 5′- and 3′-end signals were observed in the 5′ UTR of *rny*.

Using these data, we first identified putative positions of cleavage within transcripts whose abundance is known to be regulated by RNA endonuclease activity. Recent work has shown that the RNA decay machinery in *B. subtilis* is subject to extensive autoregulation and cross-regulation [15,19,50], and indeed multiple putative positions of endoribonucleolytic cleavage are detected within the transcripts encoding RNases Y, III, and the Y-complex, a complex of three small proteins (YlbF, YmcA, and YaaT) that modulates RNase Y activity [51,52] (Fig. 2, Supplementary Fig. S2). Further, a number of additional transcripts, including that encoding sporulation master regulator SinR, have been experimentally characterized as stabilized upon deletion of RNase Y [10,51]. Paired peaks are clearly visible within each of these transcripts (Fig. 2, Supplementary Fig. S2). Taken together, our ability to robustly identify known and previously unknown positions of cleavage suggests that stabilized end-sequencing provide a high-resolution, genome-wide view of RNA endonuclease activity in *B. subtilis*.

### Systematic identification of positions of endoribonuclease cleavage

To systematically identify putative positions of endonuclease cleavage, we implemented a computational approach to call abutting pairs of peaks in 3′ and 5′-end sequencing across our 4-exo and RNase J1 knockout data, respectively (Supplementary Fig. S3A). We first assessed the signal at each position relative to the average in a local window, calling positions exceeding a signal-to-background threshold as peaks. Positions for which a 5′ peak is called 1-nt downstream of a 3′ peak are called as peak pairs and represent putative sites of endoribonucleolytic activity. To restrict our analysis to mRNAs, we focused on positions within 500 nt of a coding region. This approach reveals 1966 putative positions of cleavage in mRNAs. It should be noted that we are unable to identify cleavage events proximal to RNA ends, because short RNA decay fragments (<35 nt) are excluded during 5′-end sequencing library preparation (see Materials and methods).

To assign putative cleavage events to specific endoribonucleases, we identified the positions that have diminished end-signals when an endoribonuclease is knocked out (Supplementary Fig. S3B). To account for changes in expression caused by endoribonuclease knockout, we first normalized end-sequencing signals to local RNA abundance measured by Rend-seq in the same strain. We then quantified the sensitivity of the expression-normalized signal to endoribonuclease knockout by computing the ratio of 5′-end signals (in the absence of RNase J1) between strains with and without the endoribonuclease (Materials and methods). This ‘sensitivity’ score will be elevated for positions cleaved by the corresponding endoribonuclease, as was previously shown by DiChiara *et al.* [19].

### *B. subtilis* RNase III cleaves within long-range intramolecular structures

We first characterized the cleavage profile of RNase III, motivated by reports of unexpected cleavage patterns in *B. subtilis* [19]. RNase III plays well-characterized roles in noncoding RNA processing [19,53,54] and mRNA regulation [55–57]. Several potential mRNA targets of this enzyme have been proposed, but only a small number of cleavage sites in these mRNAs have been identified [19,27]. Intriguingly, such cleavage sites often did not appear as pairs at a defined 2-nt spacing within RNA duplexes, contrasting with the hallmark of RNase III activity in *E. coli* [26]. We reasoned that a more systematic, high-resolution mapping of RNase III cleavage sites in *B. subtilis* will help resolve this issue.

**Figure 3.**
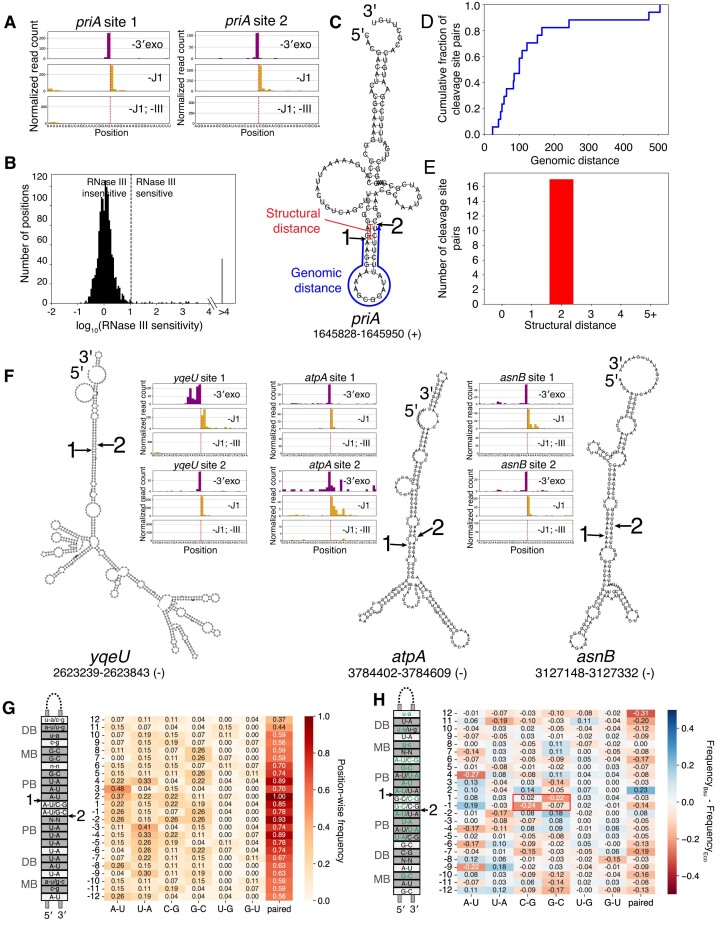
*B. subtilis* RNase III cleaves within long-range intramolecular secondary structures (**A**) Identification of RNase III cleavage positions in the *priA* mRNA. 5′- and 3′-end sequencing data are shown in yellow and purple, respectively. Identified positions of cleavage are highlighted with a dotted vertical line. Plotted are reads per million CDS-mapping reads, normalized to the average 3′-mapped Rend-seq RPM in this window. (**B**) Results of systematic identification of RNase III sites. Histogram shows distribution of endonuclease sensitivities for called peak pairs in 3′/5′-end sequencing of exoribonuclease knockouts (see Supplementary Fig. S3 and Materials and methods), with black dashed line indicating threshold for calling dependence on RNase III. 73 sites exceeded this defined threshold. For 46 sites we were unable to calculate a sensitivity score due to an absence of 5′-end sequencing counts in our knockout. These sites are called as RNase III sensitive and are counted within the “>4” bin of the histogram. (**C**) Predicted secondary structure of the sequence surrounding the identified RNase III sites within the *priA* mRNA. Positions of cleavage are indicated with arrows, with numbers corresponding to those in (**A**). Structural distance between these cleavage positions, defined as the length of the 3′ overhang generated by cleavage, is annotated in red. Genomic distance, defined as the distance between the cleavage sites in the primary sequence, is annotated in blue. (**D**) Genomic distance (as defined in C) between pairs of unambiguous RNase III cleavage positions identified within 1 kb of one another and predicted to fall on opposite sides of an RNA stem with a 2-bp spacing between cleavage positions (34 of 73 positions). (**E**) Structural distance (as defined in C) generated by RNase III cleavage at both positions within the pairs of cleavage positions plotted in (D). (**F**) Predicted secondary structures of the identified RNase III sites within the mRNAs encoding the *yqeU*, *atpA*, and *asnB* genes. Insets are plotted as in (A). (**G**) Consensus sequence-structure motif of RNase III targets in *B. subtilis*. The predicted structures for each pair of RNase III-sensitive cleavage sites that generate a 2-nt 3′ overhang were aligned to the positions of cleavage, “1” and “2”. The position-wise frequency of each type of base-pairing interaction is shown. Note that these frequencies sum to the fraction of sites paired at each position. The most frequent base pair at each position is shown in the schematic on the left, with lower-case characters used to designate positions for which the most frequent base pair occurs < 20% of the time. When two base pair frequencies are within 0.02 of one another, both are separated by a slash (e.g. A-U/G-C). N-N indicates 3+ base pairs are most frequent. The grey regions labeled “PB,” “MB,” and “DB” correspond to the proximal, middle, and distal boxes [59,60]. (**H**) Comparison of base pairing frequencies between *B. subtilis* and *E. coli* [26]. The consensus *E. coli* sequence-structure motif is shown on the left with top base pairs at each position which are shared between *B. subtilis* and *E. coli* highlighted in green. Red box highlights the decrease in C-G or G-C pairing in *B. subtilis* relative to *E. coli*.

Our stabilized end-sequencing data revealed 73 RNase III cleavage sites located on 45 mRNAs (Supplementary Table S3). These sites include all RNase III sites highlighted in Fig. 1 and Supplementary Fig. S1, and constitute a small fraction of the putative endonuclease cleavage sites we identified (5%) in the background strain (phage-cured [58]), consistent with the notion that RNase III targets a small subset of mRNAs (Fig. 3B). Despite their sparseness, these sites are often present in the same mRNAs as other RNase III sites (48 of 73) (Fig. 3A, Supplementary Fig. S1, and Supplementary Table S3). These results suggest that *B. subtilis* RNase III acts in a manner similar to the *E. coli* RNase III in cutting a pair of positions in RNA duplexes.

Indeed, of the 46 of these co-occurring cleavage sites that occur within 1 kb of each other, a large majority (38 of 46) are predicted to reside in RNA secondary structures. The extent of secondary structures is substantial, with up to 505 nt between cleavage sites (Fig. 3D). Despite the large contour length, the cleavage positions are almost always (34 of 38) separated by 2 bp on the opposite sides of RNA duplexes, which range in length from 10 to 36 bp (Fig. 3C, E, and F). Four of the 38 co-occurring sites are cleaved at two adjacent positions, which may still result from the 2-bp rule on individual RNA molecules but are difficult to resolve in our ensemble measurement. It is possible that the sites that do not form a clear intra-molecular duplex (35 of 73) participate in yet-unidentified inter-molecular pairing.

We next sought to determine what sequence features within these secondary structures might be recognized by RNase III. To increase the number of RNase III sites used in this analysis, we used a more permissive threshold on 5′-end sequencing depth (Materials and methods, Supplementary Tables S5, S6, Supplementary Fig. S3C and E), restricting our analysis to pairs of such putative cleavage positions predicted to pair with 2-bp spacing. Using this approach, we identified an additional 10 pairs of RNase III cleavage sites (27 total). The genomic distance between these sites is consistent with those initially identified (Supplementary Fig. S3D).

From these pairs of RNase III cleavage sites, we calculated the position-wise sequence content along the predicted RNA duplex, as well as the fraction of sites for which each position is paired, using the two cleavage positions as our reference points (Fig. 3G). Through this, we generated a *B. subtilis* RNase III sequence-structure motif as has previously been reported for *E. coli* [26]. Like the *E. coli* homolog (Fig. 3H), the positions surrounding *B. subtilis* RNase III cleavage sites appear to be consistently paired, with the probability of pairing increasing towards the cleavage positions (reaching > 90% at the -2/+2 positions) (Fig. 3G). Similarly, a number of sequence features appear to be recognized similarly by these enzymes, including position-specific preferences for A-U and G-C base-pairing within the proximal box (PB) and an increased occurrence of U-G wobble pairing within the distal box (DB) [26,61]. At each position from -5 to +6, at least one of the maximum-frequency base pairs is conserved between these species (Fig. 3H, green text). In contrast to *E. coli*, however, the base pairs between cleavage positions do not demonstrate a clear preference for C-G/G-C pairing in our data [26] (Fig. 3H, red box).

Together, these findings suggest that the *B. subtilis* RNase III displays similar substrate preferences to its evolutionarily distant *E. coli* homolog [26,61]. Furthermore, the presence of extensive long-range RNA secondary structure for RNase III targets suggests that mRNA base pairing across hundreds of nucleotides is common in this organism and can directly influence gene expression regulation [14,62].

### Induction of EndoA activity in exoribonuclease knockouts reveals additional 5′-end trimming activity in *B. subtilis*

**Figure 4.**
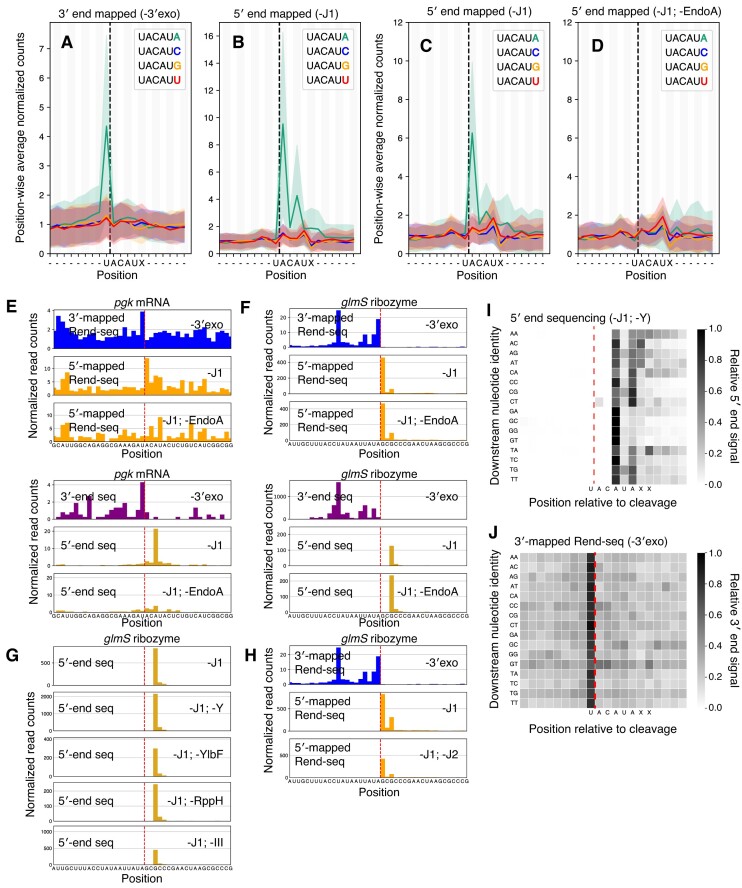
Revision of EndoA cleavage specificity and evidence for new ribonuclease activity in *B. subtilis*. (**A**and**B**) 3′ (A) and 5′ (B) mapped Rend-seq signal across all UACAU motifs in the genome, separated by downstream nucleotide. 5′-end sequencing data are derived from an RNase J1 deletion (CCB434) and 3′-end sequencing from a 4-exo knockout (CCB396). Rend-seq data at each site are normalized to first (for 3′-mapped) or last (for 5′-mapped) 8 positions within the window and a position-wise mean and standard deviation (shaded interval) are calculated with 90% winsorization. Motif instances with fewer than 1 read per position or 10 reads within the normalization window are not considered. 264 to 435 (A) or 292 to 455 (B) were included per motif. The dashed vertical line indicates the position of cleavage. (**C**and**D**) 5′-mapped Rend-seq signal in an RNase J1 depletion (CCB390) (C) or and RNase J1 depletion with deletion of *ndoA* (BT231) (D). Analysis performed as in (Aand B). 191 to 288 (C) or 86 to 117 (D) were considered per motif. (**E**) Rend-seq (top) and end sequencing (bottom) data at a representative EndoA cleavage site located in the gene *pgk* for a 4-exo knockout (CCB396), RNase J1 depletion (CCB390), and RNase J1 depletion with an *ndoA* knockout (BJT231). 5′-mapped data shown in yellow (5′-end sequencing) or orange (Rend-seq) and 3′-mapped data shown in purple (3′-end sequencing) or blue (Rend-seq). A dashed vertical line indicates the position of cleavage. Plotted are reads per million CDS-mapping reads. 5′/3′-end sequencing data are normalized to the average 3′-mapped Rend-seq signal in this window. (**F**) Rend-seq (top) and end sequencing (bottom) data at the *glmS* ribozyme cleavage. 5′-mapped data shown in yellow (5′-end sequencing) or orange (Rend-seq) and 3′-mapped data in purple (3′-end sequencing) or blue (Rend-seq). Strains included are identical to panel E. A dashed vertical line indicates the position of cleavage. Data are plotted as in (E). (**G**) 5′-end sequencing data at the position of *glmS* ribozyme cleavage for knockouts of additional RNA decay-associated proteins. All knockouts are coupled to either a depletion or deletion of RNase J1. Knocked out genes include *rnjA* (RNase J1, strain CCB434), *rny* (RNase Y, strain CCB760), *ylbF* (YlbF, strain BJT074), *rppH* (RppH, strain BJT129), and *rnc* (RNase III, strain BG879). Data are plotted as in (E), with the exception of *rppH*, which is normalized to an *rnjA* knockout alone, rather than the same genetic background, due to a lack of corresponding Rend-seq data. (**H**) Rend-seq data at the position of *glmS* ribozyme cleavage in a 4-exo knockout (CCB396), RNase J1 knockout (CCB434), and RNase J1 + J2 deficient strain (GLB186). A dashed vertical line indicates the position of cleavage. 3′-mapped data are shown in blue and 5′-mapped data are shown in orange. Plotted are reads per million CDS-mapping reads. (**I**and**J**) 5′-end sequencing (I) and Rend-seq 3′-mapped (J) signal across all UACAUA EndoA cleavage motifs, separated by downstream sequence. 5′-end sequencing data derived from an RNase J1 depletion with *rny* knockout (CCB760) and 3′-end sequencing derived from a 4-exo knockout (CCB396). The signal in a 20-nt window around each cleavage site was normalized to its maximal value and a 90% winsorized position-wise average was calculated across all normalized windows. UACAUA instances with corresponding local Rend-seq density less than 1 read per position were discarded. 10–54 sites (I) or 10–62 sites (J) were considered per row. The dashed vertical line indicates the position of cleavage by EndoA.

To our surprise, our Rend-seq data in all strains with an RNase J1 or 4-exo knockout showed peaks across the transcriptome within the motif U/ACAUA (/ denotes the cleavage position, Fig. 4A and B). This sequence resembles the canonical U/ACAU cleavage motif of the endonuclease EndoA, an RNA interferase of the MazF family associated with the EndoA-EndoAI type II toxin-antitoxin system [23,63]. The presence of adjacent 3′ and 5′ ends in Rend-seq data separating the first U/A dinucleotide within this motif suggests endoribonuclease activity (Fig. 4A and B), possibly through activation of this toxin in our exoribonuclease deficient strains. This U/ACAUA-specific cleavage is absent in Rend-seq data from wild-type *B. subtilis* (Supplementary Fig. S4A). Consistent with EndoA-mediated cleavage, which leaves a 5′ hydroxyl group [23,46], we did not observe corresponding signals in our 5′ monophosphate-specific end sequencing data (Fig. 4E). Because Rend-seq captures RNA 5′ ends through cDNA circularization rather than RNA ligation, it is agnostic to the 5′ moiety generated following cleavage [36]. This cleavage signature was fully ablated upon deletion of the EndoA-encoding gene *ndoA* (Fig. 4C and D). Furthermore, consistent with recent indications that EndoA recognizes a 6-mer rather than 5-mer motif [64], we found that only U/ACAU followed by an adenosine leads to cleavage (Fig. 4A and B).

Though stabilized end-sequencing missed the hydroxylated 5′ ends generated by EndoA, it captured unexpected peaks 2-nt downstream of canonical EndoA cleavage positions (Fig. 4E). These 5′ monophosphorylated ends only appear when EndoA is present (Fig. 4D and E), suggesting that 2 nt are removed following cleavage by EndoA. To test if this trimming activity is general against all 5′ hydroxyl groups, we looked at the self-cleaving *glmS* ribozyme, whose 5′ hydroxylated downstream cleavage product is degraded by RNase J1 [65,66]. Stabilized end-sequencing shows a clear 5′ monophosphorylated peak 2-nt downstream of the site of *glmS* self-cleavage, even in the absence of EndoA (Fig. 4F). The trimming activity is also independent of RNases Y, III, J1, J2, the Y-complex constituent YlbF, or the RNA pyrophosphohydrolase RppH (Fig. 4G and H).

We observed multiple rounds of 2-nt trimming at some, but not all, EndoA cleavage sites, suggesting sequence specificity (Supplementary Fig. S4B and C)., We therefore examined the downstream nucleotide content following the U/ACAUA EndoA motif (Fig. 4I), focusing on our *rny* knockout and RNase J1 depletion strain. This strain displays particularly clear EndoA activity, likely driven by further stabilization of EndoA cleavage products, though EndoA activity may also be increased. For EndoA sites followed by a U or C, monophosphorylated 5′ ends are found 2- and 4-nt downstream of the initial cleavage, indicating discrete trimming (Fig. 4I). For motifs followed by an A (particularly by AA), additional 5′ ends are also generated five or more nucleotides downstream of the initial cleavage. By contrast, motifs followed by a G only produce 5′ ends at 2-nt downstream. These 5′ ends do not indicate an altered EndoA cleavage position, as the 3′-mapped Rend-seq density is independent of sequence context (Fig. 4J). These data are consistent with successive rounds of nucleotide removal from RNA 5′ ends following endoribonucleolytic cleavage, with a preference for A-rich and against G-rich sequences. Differential stability following EndoA cleavage and 5′-end trimming may also influence the apparent sequence preferences we observe, however, and further work is required to reveal the identity and mode of action of this enzyme.

Cleavage 2- or 4-nt downstream of the canonical EndoA cleavage site coincides with the U/A and C/A dinucleotides that are recognized by the common laboratory contaminant RNase A. Several lines of evidence argue against such a contaminant generating the trimming behavior we characterize in this work. First, RNase A cleavage generates RNA termini with 5′ hydroxyl or 3′ phosphate groups, similar to EndoA [67]. These ends cannot be captured by our RNA 5′ and 3′-end sequencing approaches (Fig. 4E). Second, the 5′ trimming activity observed at *glmS* occurs within a C/G dinucleotide, suggesting that this activity is not restricted to U/A and C/A dinucleotides. Finally, 5′-end trimming is observed across many replicate measurements of strains lacking RNase J1.

### Determinants of cleavage by RNase Y

**Figure 5.**
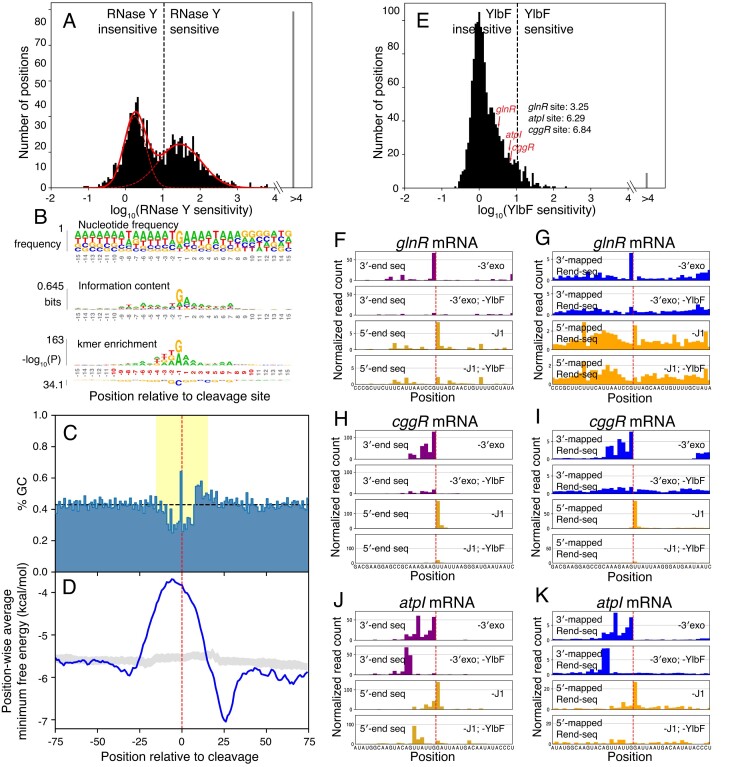
Sequence and structural features of putative RNase Y substrates. (**A**) Identification of putative RNase Y cleavage sites. Histogram shows endonuclease sensitivities for called peak pairs in 3′/5′-end sequencing of exoribonuclease knockouts (see Supplementary Fig. S3 and Materials and methods). Solid red line shows fit of two summed Gaussian distributions to log-transformed data; dashed red lines represent component distributions. The dashed vertical line indicates the lowest endonuclease sensitivity value (10.69) for which 95% of the Gaussian sum is derived from the right constituent distribution. Above this value sites are considered dependent on RNase Y. 669 of 1428 considered putative cleavage signatures exceed this threshold. Ninety-nine sites lacked 5′-end sequencing counts in the knockout needed to calculate a sensitivity score, and were instead deemed RNase Y sensitive and grouped within the “>4” bin. (**B**) Local sequence context around positions of RNase Y cleavage (*N* = 669). Nucleotide frequency (top), information content (middle) and k-mer (bottom) logos in a 30 nt window around called RNase Y sites. In k-mer logo, the most enriched and depleted k-mers are shown above and below, respectively. Significant positions (Bonferroni corrected *P*< 0.01, one-sided binomial test), are highlighted in red. (**C**) Local GC content around positions of RNase Y cleavage (*N* = 500). Sites near transcript boundaries are omitted (see Materials and methods). A dashed horizontal line indicates average %GC across *B. subtilis* genome and a dashed vertical line indicates position of cleavage. Yellow region indicates positions shown in (B**)**. (**D**) Predicted folding near RNase Y cleavage positions (*N* = 500). Sites near transcript boundaries are omitted as for (C). Blue line shows position-wise average MFE predicted by RNAfold for 40-mers tiling cleavage sites. Grey band indicates per-position interquartile range MFEs from folding randomly sampled GA-dinucleotide centered windows within coding regions, excluding putative cleavage positions (see Materials and methods). A dashed vertical line indicates putative position of cleavage. (**E**) Identification of YlbF-dependent endoribonuclease cleavage sites. Histogram shows endonuclease sensitivities for called peak pairs in stabilized end-sequencing (see Supplementary Fig. S3 and Materials and methods). The dashed vertical line denotes threshold used to call RNase Y sites. Arrows indicate endonuclease sensitivity values of canonical processing sites within the *cggR*, *glnR*, and *atpI* transcripts. Nine sites lacked 5′-end sequencing counts in the knockout needed to calculate a sensitivity score and were instead deemed YlbF sensitive and grouped in the “>4” bin. (**F**-**K**) End mapping data showing evidence for inefficient but detectable endoribonucleolytic cleavage at canonical RNase Y processing sites within the transcripts encoding *glnR* (Fand G), *cggR* (Hand I), and *atpI* (Jand K) in the absence of YlbF. (F, H, and J) show 5′/3′-end sequencing and (G, I, and K) show Rend-seq. 5′-mapped data shown in yellow (5′-end sequencing) or orange (Rend-seq) and 3′-mapped data shown in purple (3′-end sequencing) or blue (Rend-seq). A dashed vertical line indicates positions of endoribonucleolytic cleavage. Plotted are reads per million CDS-mapping reads. 5′/3′-end sequencing data are normalized to the average 3′-mapped Rend-seq signal in this window.

Though RNase Y is thought to be the primary decay-initiating endoribonuclease in *B. subtilis*, few cleavage positions have been mapped in this bacterium, and what specifies RNase Y cleavage is poorly understood [13]. To define the substrate features driving RNase Y cleavage, we sought to identify putative cleavage sites that are dependent on the presence of this enzyme. For the 1461 putative endonuclease cleavage positions identified in our W168 background, we calculated their RNase Y sensitivity (Materials and methods). This metric was bimodally distributed, and any cleavage signature with a sensitivity value over 10.69 was called as RNase Y dependent based on a double-Gaussian fit of these data (Fig. 5A). Using this approach, we identified 669 cleavage sites across 397 genes that can confidently assigned as RNase Y sites (Supplementary Table S4), including 4/8 sites highlighted in Fig. 1 and Supplementary Fig. S1 (missed sites explored in Discussion). The remaining sites could be cleaved by other endoribonucleases, such as YloC, or are not sufficiently expressed in the RNase Y knockout strain.

Our putative RNase Y sites shed light on the substrate features that guide RNase Y activity. Frequency and information content logos suggest a preference for cleavage downstream of a purine, particularly a guanosine, and upstream of an adenosine (Fig. 5B, top, middle). Similarly, considering k-mers of length ≤4, there is most significant enrichment for the dinucleotide GA spanning the cleavage site, with an additional preference for uridine in the -2 and -3 positions (Fig. 5B, bottom). This observed specificity is unlikely to reflect biases in our methodology; identification of cleavage sites requires concordance between 3′- and 5′-end sequencing datasets generated through distinct enzymatic reactions and adapter sequences. This sequence preference is additionally consistent with several previously mapped cleavage sites [9,14], as well as the measured preferences of RNase Y in *S. aureus* and *S. pyogenes*, despite the more limited role these enzymes play [7,25]. However, the GA dinucleotide motif is not absolutely required, as 63% of our putative RNase Y sites lack this motif.

In a broader window around cleavage sites, there is a clear depletion for GC-containing sequence (Fig. 5C) and a lower propensity to form secondary structures (Fig. 5D). Predicted folding of 40-nt RNAs surrounding positions of cleavage shows weaker structures for RNase Y sites compared to randomly sampled uncleaved GA dinucleotides (Fig. 5D). Interestingly, sequences centered downstream of this lowly-structured region appear to fold more strongly than background, suggesting an enrichment for secondary structure proximal to the position of cleavage (Fig. 5D). The downstream signal is recapitulated in the GC content (Fig. 5C), and is consistent with the influence of secondary structure on previously detected RNase Y cleavage events [9,68]. Together, these data indicate that *B. subtilis* RNase Y targets share a weak motif comprising a GA dinucleotide surrounded by AU-rich sequences that have reduced secondary structure but frequently with secondary structure further downstream.

### The complete Y-complex is not a strict requirement for RNase Y activity

Recent work has shown that the Y-complex, comprising proteins YlbF, YaaT, and YmcA, can act as a specificity factor for RNase Y for the maturation of nearly two-dozen operon mRNAs [15]. To understand if this complex is specifying the observed RNase Y cleavage events, we deleted the *ylbF* gene in both of our exonuclease-deficient backgrounds. We then repeated our Rend-seq and end-sequencing measurements, calculating the sensitivity of each putative cleavage site to YlbF (Fig. 5E). Compared to RNase Y, few sites appeared dependent on the presence of YlbF, suggesting that the Y-complex plays a limited role in RNA turnover outside of its known role in mRNA processing. Consistent with this observation, knocking out *ylbF* has a limited effect on the *B. subtilis* transcriptome (Supplementary Fig. S4D), as has been previously observed [15].

Surprisingly, canonical maturation events, such as those within the *cggR-gapA*, *glnRA*, and *atp* operons are still observed without YlbF, albeit strongly attenuated (Fig. 5E–H). Previously, the YlbF-dependence of these sites were determined using Rend-seq for exonuclease-expressing cells, which is less sensitive at detecting cleavage events. Here, by ablating exonucleases in cells lacking YlbF, we observed weak but detectable signals of cleavage products using Rend-seq, as well as unambiguous peaks in both 5′- and 3′-end sequencing (Fig. 5F–K). These results underscore the sensitivity of our approach and may suggest a revision of the role of the Y-complex: instead of being a strictly required factor for RNase Y cleavage, the Y-complex enhances these operon maturation events.

### RNase Y cleavage is specified by distributed substrate features

**Figure 6.**
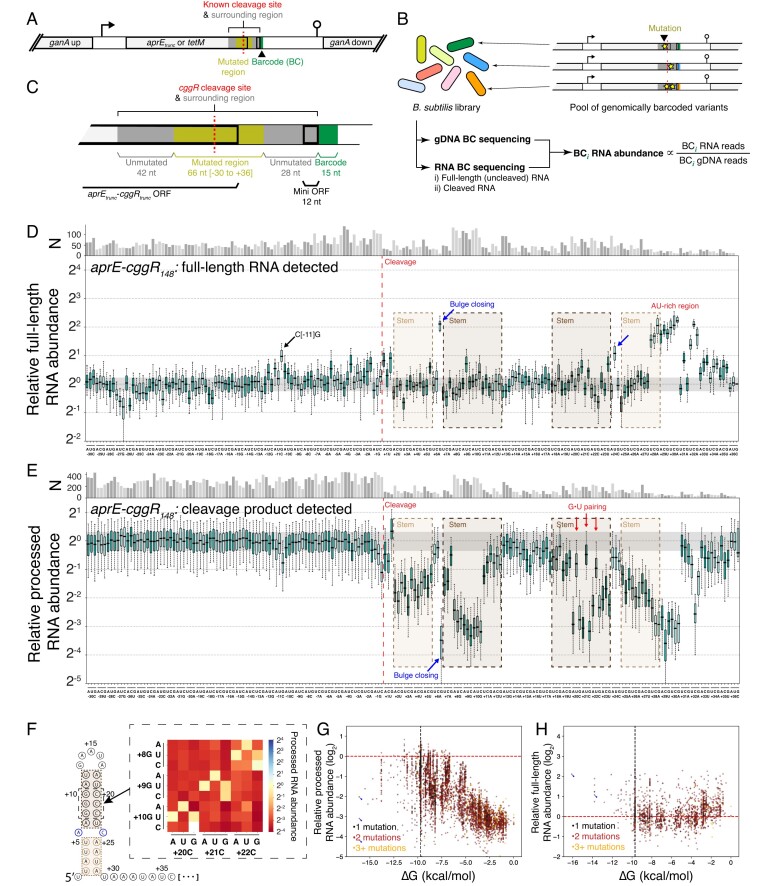
Mutational scanning of the *cggR-gapA* operon cleavage site. (**A**) Design of MPRA construct. Mutated cleavage region is indicated in grey with yellow indicating mutated positions. Variant barcode indicated in green. Translated regions are indicated with a thick border. Promoters and transcriptional terminators are indicated by bent arrows and lollipops, respectively. (**B**) Workflow for measurement of mRNA processing. Protocol begins from *B. subtilis* culture containing a pool of genetically encoded cleavage site variants, and splits into gDNA and RNA barcode quantification protocols. The relative RNA abundance is calculated as the ratio of RNA-derived to gDNA-derived barcode reads. (**C**) Schematic of the *cggR*_148_ construct inserted into the *aprE* MPRA transcript. *cggR*-derived sequence is colored grey and mutated positions yellow. Translated regions are indicated with a thick border and the variant barcode with green. (**D**) Impact of all single-nucleotide mutations on the accumulation of barcoded full-length RNA. Boxplots show variation between barcodes of identical variant sequence. Number of barcodes captured for each mutation is indicated above plot. Whiskers indicate 5^th^ and 95^th^ percentile. Grey shaded region indicates interquartile range for variants of wild-type sequence. Dashed vertical line indicates position of cleavage. Blue arrows highlight mutations that are close the bulge in the *cggR* downstream hairpin (illustrated in F). Four variants (≤1 per mutation) have a value of zero are thus not visualized. (**E**) Impact of all single-nucleotide mutations on the accumulation of barcoded cleavage product. Boxplots show variation between barcodes of identical variant sequence. Number of barcodes captured for each mutation (N) is indicated by the bar height (top). Whiskers indicate 5^th^ and 95^th^ percentile. Grey shaded region indicates interquartile range for variants of wild-type sequence. Dashed vertical line indicates position of cleavage and brown bars indicate regions predicted to pair in the formation of a downstream stem-loop structure (illustrated in F). Red arrows indicate mutations predicted to generate G•U wobble pairing, and the blue arrow highlights a mutation which corrects the bulge in this structure. Four variants (≤1 per mutation) have a value of zero are thus not visualized. (**F**) Predicted stem-loop structure at 5′-end of processed RNA. Inset shows impact of double mutants on accumulation of processed RNA. Double mutant averages calculated over any variants with the two indicated mutations. Any variants which contain additional mutations that on their own result in greater than 10% change in processed RNA accumulation are discarded. (**G**) Relationship between predicted strength of downstream secondary structure and accumulation of barcoded cleavage product. The experiment each data point is derived from is denoted with a triangle (experiment 2b, Supplementary Table S7), circle (4a), or square (6). The vertical dashed line indicates the ΔG of the unmutated sequence and horizontal dashed line indicates the median abundance of unmutated variants. Five variants fall above the bounds of this plot (Supplementary Table S8). Blue arrows indicate bulge-closing mutation highlighted in (E). (**H**) Relationship between predicted strength of downstream secondary structure and accumulation of barcoded full-length RNA. All data derived from experiment 2a (Supplementary Table S7). The vertical dashed line indicates the ΔG of the unmutated sequence and horizontal line indicates the median abundance of unmutated variants. Five variants fall above the bounds of this plot (Supplementary Table S9). Blue arrows indicate bulge-closing mutations highlighted in (D).

Our census of RNase Y cleavage events showed a degenerate motif, which is insufficient to explain its precise site selection. To identify additional sequence or structural determinants, we developed an MPRA for high-throughput measurement of cleavage efficiency across 10^4^–10^5^ mutated cleavage sites (Fig. 6, Supplementary Table S7). The reporter consists of a >100-nt region surrounding a cleavage site inserted into a stable mRNA encoding the gene *aprE*, truncated to lack an active site (Fig. 6A). These constructs are integrated at the *ganA* locus and contain a randomized 15-nt barcode, allowing relative quantification of each variant's expression by RNA and DNA sequencing (Fig. 6B). To do so, we purified and reverse-transcribed RNA from exponentially-growing cells, using gel purification to separate cleaved from full-length RNA. This allows specific detection of each isoform. A reduction in cleavage should promote accumulation of the full-length RNA and concomitant decrease in cleaved RNA, such that a mutated variant's barcode abundance in uncleaved RNA, normalized to its frequency in the pool by gDNA sequencing, provides a readout of its cleavage efficiency (Fig. 6B).

We initially focused our MPRA measurements on a site within the *cggR* gene of the glycolytic operon [69,70] (Fig. 5F and G). This cleavage produces a downstream product, which includes the *gapA* gene, that is thought to be stabilized by an RNA hairpin structure at the cleaved end (henceforth referred to as the downstream hairpin) [70]. Whether proximal structure also plays a major role in specifying the cleavage site by *B. subtilis* RNase Y remains unknown. To capture surrounding sequences that contribute to this cleavage, we inserted 148 bp of this sequence into our reporter (Fig. 6C). The site of cleavage, normally within the *cggR* open reading frame, is translated in the reading frame of the reporter, followed by a stop codon and a short open reading frame derived from the start of the downstream *gapA* gene (Fig. 6C). Using this approach, we characterized the effect of mutations from positions −30 to +36 relative to the cleavage. After sequencing, 91 580 uniquely barcoded variants were detected across all experiments. These data cover virtually all possible single-nucleotide changes (Fig. 6D) and 32% and 44% of all double-nucleotide changes across experiments detecting full-length and cleaved RNAs, respectively (Supplementary Fig. S5A and B). For each mutant, we average the effects over different barcode variants (median 44 barcodes for single-nucleotide changes) to reduce noise and barcode-driven variation (Supplementary Fig. S5C and D).

Mutations across a wide region surrounding the *cggR* cleavage site showed modest but detectable impacts on steady-state full-length mRNA abundance (Fig. 6D). Between −30 and +1 nt relative to the cleavage site, few mutations appear to reduce cleavage efficiency. The strongest effects are seen at the cytosine at position −11 (denoted as C[−11]), and the effect sizes are at most twofold. Interestingly, mutations of G at position −1 showed no statistically significant effect, even though it is the strongest consensus among RNase Y cleavage sites, a result consistent with our finding that 37% of the RNase Y cleavage sites detected do not have a guanine at this position (Fig. 5B). Downstream of the cleavage site, mutations in the hairpin region also produced limited effects on full-length RNA levels for both single substitutions (Fig. 6D) and combinations which further destabilize the hairpin (Fig. 6H). Surprisingly, the only mutations in this structured region that reduce cleavage are substitutions in a bulge of the downstream hairpin (Fig. 6F), including an A[+6]G substitution (fourfold) and a C[+24]U substitution (twofold) (Fig. 6D), both of which complete the hairpin. This suggests that the downstream hairpin is not essential for cleavage, and that a stronger hairpin can in fact negatively impact cleavage.

Immediately downstream of the hairpin, an AU-rich region appears to play a role in cleavage. Mutations to AUAAAUAU located at position +28 to +35 lead to increases in full-length RNA level by up to 4-fold. In the folded RNA, this region is brought to be adjacent to the cleavage site by the intervening hairpin (Fig. 6F). Although it is plausible that such spatial proximity enables the distal region to recruit RNase Y or facilitate its activity, the fact that mutations in the hairpin region have no detectable effects suggests that other mechanisms may be at play.

To further elucidate the role of the downstream hairpin, we tested the hypothesis that this secondary structure mainly serves as a stabilizer for the cleaved product by preventing exoribonucleolytic decay [70]. This role is distinct from the model for the *S. aureus cggR-gapA* operon, where the downstream hairpin has been suggested to specify RNase Y cleavage sites [68,71]. To distinguish these roles, we conducted deep mutational scanning on the accumulation of processed mRNA, measuring 349 266 mutant variants in total. Unlike full-length mRNA, the level of the downstream processed mRNA should depend on both cleavage efficiency and its own half-life post-cleavage.

Most mutations that increased full-length mRNA level resulted in reduced accumulation of the cleavage product, but not vice versa (Fig. 6E). The effects of C[−11] mutations, bulge-closing mutations, and the distal AU-rich region are recapitulated in downstream cleavage product levels. However, in contrast to the full-length RNA, mutations in the downstream hairpin strongly suppressed cleavage product accumulation. Consistent with secondary structure playing a role, double-substitution variants that complement these mutations (Fig. 6F) have dramatically reduced effect sizes, as are stem mutations that maintain base pairing (through wobble interactions, Fig. 6E, red arrows). Considering all variants, we found that a stronger downstream hairpin is predictive of the cleaved RNA level (Fig. 6G), in stark contrast with a lack of correlation for full-length RNA (Fig. 6H). Finally, we measured cleaved RNA abundance in the context of RNase J1 depletion, and found that, apart from closing the bulge, downstream stem mutations no longer measurably impact cleaved RNA levels without exoribonucleolytic activity (Supplementary Fig. S6A). Together, these results suggest that the downstream hairpin primarily dictates cleavage product abundance rather than efficiency of RNase Y cleavage.

To test if the role proximal secondary structure is specific to the *cggR* operon, we analyzed a second site within the *glnRA* operon (Supplementary Fig. S7A). This cleavage also occurs after the operon's first gene (*glnR*) and a stabilizing stem-loop structure is predicted to form downstream (Supplementary Fig. S7B). We quantified cleaved RNA for 54 431 variants, mutated at positions −5 to +31 relative to the cleavage. As before, single (Supplementary Fig. S7C) and higher-order (Supplementary Fig. S7D, Supplementary Table S10) stem substitutions attenuated the accumulation of the cleavage product (up to approximately 4-fold). Disruption of the stem did not stabilize the full-length RNA (Supplementary Fig. S7E and F). Similarly structured mRNA processing sites may thus function similarly to *cggR*. To assess the context-dependence of these features, we placed the *cggR* site into an alternative reporter construct encoding the gene *tetM*. Key effects were consistent, but the *tetM* reporter showed additional deleterious substitutions in C[−19]G and A[−6]G (Supplementary Fig. S6B). C[−19] is situated within the *cggR* reading frame in a purine-rich region, AGA **C** GAAGGAG. The deleterious effects were still observed when we introduced an upstream stop codon, suggesting that these substitutions do not act through ribosomes slowed by a Shine-Dalgarno-like sequence, but rather through other context-specific means (Supplementary Fig. S6C). These results reinforce that RNase Y cleavage determinants are spread across a broad region of the mRNA.

## Discussion

**Figure 7.**
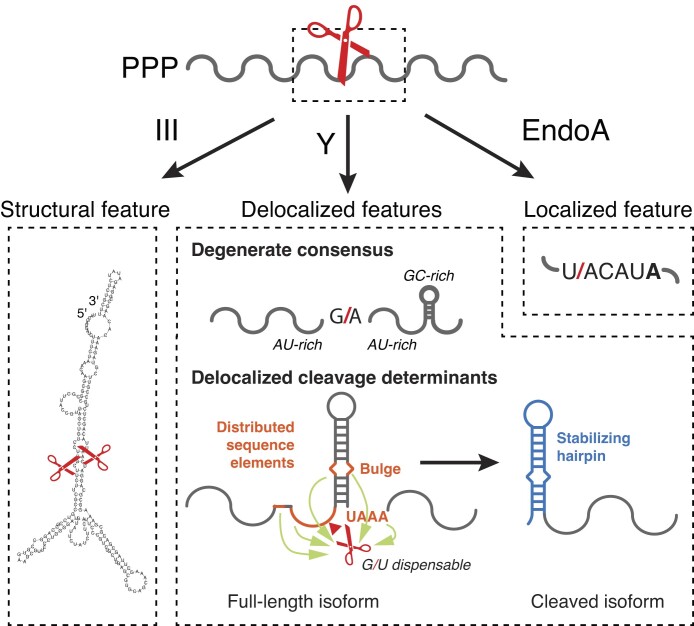
Determinants of endoribonuclease cleavage identified in this work. (**A**) Summary of the identified cleavage determinants of the three endoribonucleases profiled in this work. RNase III cleaves within double-stranded RNA sequences, including many duplexes formed through long range (>100 nt) intramolecular interactions, leaving a 2-nt 3′ overhang (left, ‘III’). EndoA cleaves specifically at a UACAUA primary sequence motif (right, ‘EndoA’). RNase Y cleavage appears to target AU-rich regions of low secondary structure, preferring cleavage within a G/A dinucleotide (center, ‘Y’). Interrogation of the cleavage sites within *cggR* and *glnR* suggest that a -1G is dispensable for RNase Y cleavage, and for a given site, the sequence and structural elements that drive cleavage ("distributed sequence elements") may be distributed over many tens of nucleotides. Further, for some mRNA processing sites such as those in *cggR* or *gapA*, a downstream stem-loop structure ("stabilizing hairpin") appears to primarily drive stabilization of the cleaved isoform.

In this work, we present the first genome-scale map of *B. subtilis* endoribonuclease cleavages at single-nucleotide resolution. Using stabilized end-sequencing, we identified over a thousand putative cleavage events, revealing substrate preferences of the central endonucleases involved in *B. subtilis* mRNA decay (Fig. 7). This work captured the importance of long-range RNA base pairing for RNase III cleavage and confirmed that the toxin EndoA recognizes a hexameric rather than pentameric motif. We additionally uncovered evidence for and potential specificity of a new ribonucleolytic activity previously unknown in *B. subtilis*. Finally, we identified hundreds of new positions of cleavage by the core endonuclease driving mRNA decay, RNase Y. Combining these data with a new MPRA, we demonstrate that RNase Y cleavage is specified by features distributed over many positions across regions tens of nucleotides in length. This stands in stark contrast to EndoA, which recognizes a strict consensus cleavage sequence, and RNase III, which has a strongly stereotyped cleavage behavior within secondary structures.

The distributed suite of features that contribute to RNase Y processing of *cggR* helps explain the highly degenerate motif that we observed through global cleavage site mapping. The ability for features distal in primary sequence to influence cleavage may be mediated by RNA folding or by higher-order RNA–protein and protein–protein interactions. As such, the particular positions recognized at each site may be variable and highly context-specific. Interestingly, a common feature we identified between many RNase Y sites, the presence of a -1G, only have a modest effect on the accumulation of processed *cggR* RNA in our reporter system, if any. This discrepancy may suggest a similar model to what is observed in in *S. aureus*, where an upstream guanosine fine-tunes the position of cleavage but is not required for cleavage activity [71].

The absence of a clear consensus motif specifying RNase Y activity echoes findings for RNase E in *E. coli* [24,72,73]. The code for RNA decay appears to fundamentally differ from transcription and translation, whose rates are influenced by well-defined interactions between the gene expression machinery and localized sequence features within promoters or ribosome binding sites [1]. Instead, decay-initiating RNases such as RNase Y appear to be strongly influenced by features distal to the position of cleavage, possibly facilitated by RNA secondary structure and RNA-protein interactions. mRNA stability may also be strongly influenced by features extrinsic to primary RNA sequence. Decay can occur concurrently with and may be influenced by transcription and translation [74,75], and the membrane localization of decay machinery in bacterial cells also means that protein binding and RNA localization may have a strong influence on RNA stability [6,76,77]. Robust prediction of endoribonuclease cleavage from primary sequence will thus likely require integration of more modes of measurement and interrogation of more cleavage sites than our current end-mapping approach and MPRA allow.

Although the presented global map of endonuclease activity expands our list of known endoribonuclease cleavage positions by orders of magnitude, we do not expect it to be comprehensive. Indeed, we only capture 8/12 known RNase III/Y cleavage sites highlighted in Supplementary Fig. S1. First, our end-sequencing protocol is unable to detect cleavage sites within 15 nt of a 5′ end or 40 nt of a 3′ end of a transcription unit. Additionally, while all known exoribonuclease activity in one direction is ablated in each of our experiments, secondary endoribonuclease cleavages may obscure a particular site by either generating sufficient background signal to prevent its automatic detection or by allowing unperturbed exoribonucleases acting in the opposite direction to destabilize the cleavage products, rendering them undetectable. Finally, unknown exoribonuclease activities may cause shifting of 3′ or 5′ ends, separating the peak pairs and obscuring potential cleavage positions (as seen in Fig. 4). Therefore, the absence of signal in these data may not necessarily indicate that turnover of a particular RNA is independent of endoribonucleolytic cleavage.

Unexpectedly, our analysis of EndoA cleavage sites and the *glmS* ribozyme revealed a previously uncharacterized *B. subtilis* 5′-end trimming activity. We have confirmed that this activity is not dependent on RNases Y, III, J1, J2, EndoA, or RppH. Whether this nuclease acts in a 5′-3′ exonucleolytic or endoribonucleolytic manner remains to be determined. Further work is additionally necessary to understand whether this activity facilitates RNase J1 cleavage at substrates with a 5′ hydroxyl moiety, influencing how the *glmS* ribozyme regulates gene expression. Determining what gene encodes this activity is thus of great interest.

Our finding that the full Y-complex is not strictly required for mRNA processing further complicates the role of this complex in RNA maturation and decay. Although our data support that loss of YlbF strongly attenuates cleavage at the operons the Y complex canonically regulates, persistence of weak cleavage signatures suggests that YlbF is not strictly required for RNase Y activity at these sites. This residual cleavage may represent dispensability of the Y-complex as a whole or of YlbF for Y-complex activity. Indeed, Dubnau *et al.* have suggested that, of the Y-complex members, only YaaT (RicT) stably associates with RNase Y to form the functional complex [52]. It remains to be tested whether deletion of YaaT eliminates the low-level cleavage activity we detect in the absence of YlbF.

Mutational scanning of the *cggR* mRNA processing site revealed that downstream secondary structure primarily influences *cggR* operon stoichiometry through stabilization of the product of cleavage rather than driving cleavage itself. While consistent with early studies in *B. subtilis* [70], this result appears to contradict findings that the presence of a downstream hairpin is a key driver of *S. aureus* RNase Y cleavage at many sites [68,71]. It is worth noting, however, that *S. aureus* RNase Y targets fewer RNAs than its *B. subtilis* homolog and its regulation may differ [7,78]. Additionally, the single base substitutions investigated here are a milder perturbation than used in other studies of the *cggR* operon [71], and when secondary structure is strongly ablated in our experiments, a mild stabilization of the full-length construct is observed (Fig. 6H). It is thus possible that although the effect of secondary structure on cleavage product stability is more pronounced, downstream RNA structure is playing a dual role in this system.

Our approach can be generalized to other bacteria for which exonuclease activity can be ablated or transiently depleted, representing a new approach to discover factors influencing RNA decay. Additionally, application of this approach to conditions beyond exponential growth in rich medium will provide insight into the regulation of endonuclease activity and RNA decay more broadly. The high-resolution picture of RNA decay initiation provided by this approach, particularly when integrated with future measurements of RNA half-life, will inform quantitative predictions of RNA stability and gene expression levels from primary sequence in Gram-positive bacteria.

## Supplementary Material

gkaf030_Supplemental_Files

## Data Availability

All sequencing data generated in this manuscript are available at the Gene Expression Omnibus with the accession number GSE275083. Scripts used in processing stabilized end-sequencing and MPRA data are available at https://doi.org/10.5281/zenodo.14574885.

## Notes

Present address: department of systems biology, harvard medical school, boston, ma 02115, usa

Present address: Département de Biochimie et de Médecine Moléculaire Université de Montréal, C.P. 6128, Station Centre-Ville, Montréal (Québec), H3C 3J7, Canada

## References

[1] Taggart JC, Lalanne JB, Li GW. Quantitative control for stoichiometric protein synthesis. Annu Rev Microbiol. 2021; 75:243–67. 34343023 10.1146/annurev-micro-041921-012646 PMC8720029

[2] Brewster RC, Jones DL, Phillips R. Tuning promoter strength through RNA polymerase binding site design in *Escherichia coli*. PLoS Comput Biol. 2012; 8:e1002811. 23271961 10.1371/journal.pcbi.1002811 PMC3521663

[3] Urtecho G, Tripp AD, Insigne KD et al. Systematic dissection of sequence elements controlling σ70 promoters using a genomically encoded multiplexed reporter assay in Escherichia coli. Biochemistry. 2019; 58:1539–51. 29388765 10.1021/acs.biochem.7b01069 PMC6389444

[4] Salis HM, Mirsky EA, Voigt CA. Automated design of synthetic ribosome binding sites to control protein expression. Nat Biotechnol. 2009; 27:946–50. 19801975 10.1038/nbt.1568 PMC2782888

[5] Salis HM. The ribosome binding site calculator. Methods Enzym. 2011; 498:19–42. 10.1016/B978-0-12-385120-8.00002-4 21601672

[6] Moffitt JR, Pandey S, Boettiger AN et al. Spatial organization shapes the turnover of a bacterial transcriptome. eLife. 2016; 5:e13065. 27198188 10.7554/eLife.13065 PMC4874777

[7] Khemici V, Prados J, Linder P et al. Decay-initiating endoribonucleolytic cleavage by RNase Y is kept under tight control via sequence preference and sub-cellular localisation. PLoS Genet. 2015; 11:e1005577. 26473962 10.1371/journal.pgen.1005577 PMC4608709

[8] Chen H, Shiroguchi K, Ge H et al. Genome-wide study of mRNA degradation and transcript elongation in *Escherichia coli*. Mol Syst Biol. 2015; 11:781. 25583150 10.15252/msb.20145794 PMC4332155

[9] Shahbabian K, Jamalli A, Zig L et al. RNase Y, a novel endoribonuclease, initiates riboswitch turnover in *Bacillus subtilis*. EMBO J. 2009; 28:3523–33. 19779461 10.1038/emboj.2009.283 PMC2782095

[10] Lehnik-Habrink M, Schaffer M, Mäder U et al. RNA processing in *B**acillus subtilis*: identification of targets of the essential RNase Y. Mol Microbiol. 2011; 81:1459–73. 21815947 10.1111/j.1365-2958.2011.07777.x

[11] Lehnik-Habrink M, Lewis RJ, Mäder U et al. RNA degradation in *Bacillus subtilis*: an interplay of essential endo- and exoribonucleases. Mol Microbiol. 2012; 84:1005–17. 22568516 10.1111/j.1365-2958.2012.08072.x

[12] Durand S, Gilet L, Bessières P et al. Three essential ribonucleases-RNase Y, J1, and III-control the abundance of a majority of *Ba**cillus subtilis* mRNAs. PLoS Genet. 2012; 8:e1002520. 22412379 10.1371/journal.pgen.1002520 PMC3297567

[13] Trinquier A, Durand S, Braun F et al. Regulation of RNA processing and degradation in bacteria. Biochim Biophys. 2020; 1863:194505. 10.1016/j.bbagrm.2020.194505 32061882

[14] Braun F, Durand S, Condon C. Initiating ribosomes and a 5- /3-UTR interaction control ribonuclease action to tightly couple B. subtilis hbs mRNA stability with translation. Nucleic Acids Res. 2017; 45:11386–400. 28977557 10.1093/nar/gkx793 PMC5737220

[15] DeLoughery A, Lalanne J-B, Losick R et al. Maturation of polycistronic mRNAs by the endoribonuclease RNase Y and its associated Y-complex in *Bacillus subtilis*. Proc Natl Acad Sci USA. 2018; 115:E5585–94. 29794222 10.1073/pnas.1803283115 PMC6004469

[16] Noone D, Salzberg LI, Botella E et al. A highly unstable transcript makes CwlO D, L-endopeptidase expression responsive to growth conditions in *Bacillus subtilis*. J Bacteriol. 2014; 196:237–47. 24163346 10.1128/JB.00986-13 PMC3911235

[17] Commichau FM, Rothe FM, Herzberg C et al. Novel activities of glycolytic enzymes in *Bacillus**subtilis*: interactions with essential proteins involved in mRNA processing. Mol Cell Proteomics. 2009; 8:1350–60. 19193632 10.1074/mcp.M800546-MCP200 PMC2690492

[18] Benda M, Woelfel S, Faßhauer P et al. Quasi-essentiality of RNase Y in *Bacillus subtilis* is caused by its critical role in the control of mRNA homeostasis. Nucleic Acids Res. 2021; 49:7088–102. 34157109 10.1093/nar/gkab528 PMC8266666

[19] DiChiara JM, Liu B, Figaro S et al. Mapping of internal monophosphate 5′ ends of *Bacillus subtilis* messenger RNAs and ribosomal RNAs in wild-type and ribonuclease-mutant strains. Nucleic Acids Res. 2016; 44:3373–89. 26883633 10.1093/nar/gkw073 PMC4838370

[20] Cerullo F, Filbeck S, Patil PR et al. Bacterial ribosome collision sensing by a MutS DNA repair ATPase paralogue. Nature. 2022; 603:509–14. 35264791 10.1038/s41586-022-04487-6 PMC9041291

[21] Leroy M, Piton J, Gilet L et al. Rae1/YacP, a new endoribonuclease involved in ribosome-dependent mRNA decay in *Bacillus subtilis*. EMBO J. 2017; 36:1167–81. 28363943 10.15252/embj.201796540 PMC5412759

[22] Ingle S, Chhabra S, Chen J et al. Discovery and initial characterization of YloC, a novel endoribonuclease in *Bacillus subtilis*. RNA. 2022; 28:227–38. 34815358 10.1261/rna.078962.121 PMC8906540

[23] Pellegrini O, Mathy N, Gogos A et al. The *Bacillus subtilis* ydcDE operon encodes an endoribonuclease of the MazF/PemK family and its inhibitor. Mol Microbiol. 2005; 56:1139–48. 15882409 10.1111/j.1365-2958.2005.04606.x

[24] Chao Y, Li L, Girodat D et al. In vivo cleavage map illuminates the central role of RNase E in coding and non-coding RNA pathways. Mol Cell. 2017; 65:39–51. 28061332 10.1016/j.molcel.2016.11.002 PMC5222698

[25] Broglia L, Lécrivain A, Renault TT et al. An RNA-seq based comparative approach reveals the transcriptome-wide interplay between 3’-to-5’ exoRNases and RNase Y. Nat Commun. 2020; 11:1587. 32221293 10.1038/s41467-020-15387-6 PMC7101322

[26] Altuvia Y, Bar A, Reiss N et al. In vivo cleavage rules and target repertoire of RNase III in *Escherichia coli*. Nucleic Acids Res. 2018; 46:10530–31. 30184218 10.1093/nar/gky816 PMC6212792

[27] Gordon GC, Cameron JC, Pfleger BF. RNA sequencing identifies new RNase III cleavage sites in *Escherichia coli* and reveals increased regulation of mRNA. mBio. 2017; 8:e00128-17. 28351917 10.1128/mBio.00128-17 PMC5371410

[28] Yao S, Bechhofer DH. Initiation of decay of *B**acillus subtilis* rpsO mRNA by endoribonuclease RNase Y. J Bacteriol. 2010; 192:3279–86. 20418391 10.1128/JB.00230-10 PMC2897663

[29] Bruscella P, Shahbabian K, Laalami S et al. RNase Y is responsible for uncoupling the expression of translation factor IF3 from that of the ribosomal proteins L35 and L20 in *Bacillus subtilis*. Mol Microbiol. 2011; 81:1526–41. 21843271 10.1111/j.1365-2958.2011.07793.x

[30] Parker DJ, Lalanne JB, Kimura S et al. Growth-optimized aminoacyl-tRNA synthetase levels prevent maximal tRNA charging. Cell Syst. 2020; 11:121–130. 32726597 10.1016/j.cels.2020.07.005 PMC7484455

[31] Yasbin RE, Young FE. Transduction in *Bacillus subtilis* by bacteriophage SPP1. J Virol. 1974; 14:1343–48. 4214946 10.1128/jvi.14.6.1343-1348.1974 PMC355660

[32] Britton RA, Wen T, Schaefer L et al. Maturation of the 5′ end of Bacillus subtilis 16S rRNA by the essential ribonuclease YkqC/RNase J1. Mol Microbiol. 2007; 63:127–38. 17229210 10.1111/j.1365-2958.2006.05499.x

[33] Koo BM, Kritikos G, Farelli JD et al. Construction and analysis of two genome-scale deletion libraries for *Bacillus subtilis*. Cell Syst. 2017; 4:291–305. 28189581 10.1016/j.cels.2016.12.013 PMC5400513

[34] Zhang XZ, Zhang YHP. Simple, fast and high-efficiency transformation system for directed evolution of cellulase in *Bacillus subtilis*. Microb Biotechnol. 2011; 4:98–105. 21255377 10.1111/j.1751-7915.2010.00230.x PMC3815800

[35] Stead MB, Agrawal A, Bowden KE et al. RNAsnap^TM^: a rapid, quantitative and inexpensive, method for isolating total RNA from bacteria. Nucleic Acids Res. 2012; 40:e156. 22821568 10.1093/nar/gks680 PMC3488207

[36] Lalanne JB, Taggart JC, Guo MS et al. Evolutionary convergence of pathway-specific enzyme expression stoichiometry. Cell. 2018; 173:749–61. 29606352 10.1016/j.cell.2018.03.007 PMC5978003

[37] Herzel L, Stanley JA, Yao C-C et al. Ubiquitous mRNA decay fragments in *E. coli* redefine the functional transcriptome. Nucleic Acids Res. 2022; 50:5029–46. 35524564 10.1093/nar/gkac295 PMC9122600

[38] Subtelny AO, Eichhorn SW, Chen GR et al. Poly(A)-tail profiling reveals an embryonic switch in translational control. Nature. 2014; 508:66–71. 24476825 10.1038/nature13007 PMC4086860

[39] Johnson GE, Parker DJ, Lalanne J et al. BaM-seq and TBaM-seq, highly multiplexed and targeted RNA-seq protocols for rapid, low-cost library generation from bacterial samples. 2023; 5:lqad017. 10.1093/nargab/lqad017 PMC9985320 36879903

[40] Martin M. Cutadapt removes adapter sequences from high-throughput sequencing reads. EMBnet J. 2011; 17:10–12.

[41] Langmead B, Trapnell C, Pop M et al. Ultrafast and memory-efficient alignment of short DNA sequences to the human genome. Genome Biol. 2009; 10:R25. 19261174 10.1186/gb-2009-10-3-r25 PMC2690996

[42] Lorenz R, Bernhart SH, Zu Siederdissen CH et al. ViennaRNA package 2.0. Algorithms Mol Biol. 2011; 6:26. 22115189 10.1186/1748-7188-6-26 PMC3319429

[43] Wu X, Bartel DP. kpLogo: positional k-mer analysis reveals hidden specificity in biological sequences. Nucleic Acids Res. 2017; 45:W534–38. 28460012 10.1093/nar/gkx323 PMC5570168

[44] Oussenko IA, Abe T, Ujiie H et al. Participation of 3′-to-5′ exoribonucleases in the turnover of *B**acillus subtilis* mRNA. J Bacteriol. 2005; 187:2758–67. 15805522 10.1128/JB.187.8.2758-2767.2005 PMC1070398

[45] Figaro S, Durand S, Gilet L et al. Bacillus subtilis mutants with knockouts of the genes encoding ribonucleases RNase Y and RNase J1 are viable, with major defects in cell morphology, sporulation, and competence. J Bacteriol. 2013; 195:2340–48. 23504012 10.1128/JB.00164-13 PMC3650553

[46] Bechhofer DH, Deutscher MP. Bacterial ribonucleases and their roles in RNA metabolism. Crit Rev Biochem Mol Biol. 2019; 54:242–300. 31464530 10.1080/10409238.2019.1651816 PMC6776250

[47] Dar D, Shamir M, Mellin JR et al. Term-seq reveals abundant ribo-regulation of antibiotics resistance in bacteria. Science. 2016; 352:aad9822. 27120414 10.1126/science.aad9822 PMC5756622

[48] Mandell ZF, Oshiro RT, Yakhnin AV et al. NusG is an intrinsic transcription termination factor that stimulates motility and coordinates global gene expression with NusA. eLife. 2021; 10:e61880. 33835023 10.7554/eLife.61880 PMC8060035

[49] Durand S, Braun F, Lioliou E et al. A nitric oxide regulated small RNA controls expression of genes involved in redox homeostasis in *Bacillus subtilis*. PLoS Genet. 2015; 11:e1004957. 25643072 10.1371/journal.pgen.1004957 PMC4409812

[50] Korobeinikova A, Laalami S, Berthy C et al. RNase Y autoregulates its synthesis in *Bacillus subtilis*. Microorganisms. 2023; 11:1374. 37374876 10.3390/microorganisms11061374 PMC10303841

[51] Deloughery A, Dengler V, Chai Y et al. Biofilm formation by *Bacillus subtilis* requires an endoribonuclease-containing multisubunit complex that controls mRNA levels for the matrix gene repressor SinR. Mol Microbiol. 2016; 99:425–37. 26434553 10.1111/mmi.13240 PMC4989519

[52] Dubnau E, DeSantis M, Dubnau D. Formation of a stable RNase Y-RicT (YaaT) complex requires RicA (YmcA) and RicF (YlbF). mBio. 2023; 14:e0126923. 37555678 10.1128/mbio.01269-23 PMC10470536

[53] Herskovitz MA, Bechhofer DH. Endoribonuclease RNase III is essential in *Bacillus subtilis*. Mol Microbiol. 2000; 38:1027–33. 11123676 10.1046/j.1365-2958.2000.02185.x

[54] Oguro A, Kakeshita H, Nakamura K et al. *Bacillus subtilis* RNase III cleaves both 5’- and 3’-sites of the small cytoplasmic RNA precursor. J Biol Chem. 1998; 273:19542–47. 9677377 10.1074/jbc.273.31.19542

[55] Durand S, Gilet L, Condon C. The essential function of B. subtilis RNase III is to silence foreign toxin genes. PLoS Genet. 2012; 8:e1003181. 23300471 10.1371/journal.pgen.1003181 PMC3531473

[56] Jahn N, Brantl S. One antitoxin-two functions: SR4 controls toxin mRNA decay and translation. Nucleic Acids Res. 2013; 41:9870–80. 23969414 10.1093/nar/gkt735 PMC3834814

[57] Meißner C, Jahn N, Brantl S. *In vitro* characterization of the type I toxin-antitoxin system bsrE/SR5 from *Bacillus subtilis*. J Biol Chem. 2016; 291:560–71. 26565032 10.1074/jbc.M115.697524 PMC4705377

[58] Gilet L, Dichiara JM, Figaro S et al. Small stable RNA maturation and turnover in *Bacillus subtilis*. Mol Microbiol. 2015; 95:270–82. 25402410 10.1111/mmi.12863 PMC4364036

[59] Zhang K, Nicholson AW. Regulation of ribonuclease III processing by double-helical sequence antideterminants. Proc Natl Acad Sci USA. 1997; 94:13437–41. 9391043 10.1073/pnas.94.25.13437 PMC28323

[60] Gan J, Tropea JE, Austin BP et al. Structural insight into the mechanism of double-stranded RNA processing by ribonuclease III. Cell. 2006; 124:355–66. 16439209 10.1016/j.cell.2005.11.034

[61] Pertzev AV, Nicholson AW. Characterization of RNA sequence determinants and antideterminants of processing reactivity for a minimal substrate of *Escherichia coli* ribonuclease III. Nucleic Acids Res. 2006; 34:3708–21. 16896014 10.1093/nar/gkl459 PMC1540722

[62] Ruiz de los Mozos I, Vergara-Irigaray M, Segura V et al. Base pairing interaction between 5′- and 3′-UTRs controls icaR mRNA translation in *Staphylococcus aureus*. PLoS Genet. 2013; 9:e1004001. 24367275 10.1371/journal.pgen.1004001 PMC3868564

[63] Park J-H, Yamaguchi Y, Inouye M. *Bacillus subtilis* MazF-bs (EndoA) is a UACAU-specific mRNA interferase. FEBS Lett. 2011; 585:2526–32. 21763692 10.1016/j.febslet.2011.07.008 PMC3167231

[64] Guler P, Bendori SO, Borenstein T et al. Arbitrium communication controls phage lysogeny through non-lethal modulation of a host toxin–antitoxin defence system. Nat Microbiol. 2024; 9:150–60. 38177304 10.1038/s41564-023-01551-3

[65] Collins JA, Irnov I, Baker S et al. Mechanism of mRNA destabilization by the glmS ribozyme. Genes Dev. 2007; 21:3356–68. 18079181 10.1101/gad.1605307 PMC2113035

[66] Winkler WC, Nahvi A, Roth A et al. Control of gene expression by a natural metabolite-responsive ribozyme. Nature. 2004; 428:281–86. 15029187 10.1038/nature02362

[67] Cuchillo CM, Nogués MV, Raines RT. Bovine pancreatic ribonuclease: fifty years of the first enzymatic reaction mechanism. Biochemistry. 2011; 50:7835–41. 21838247 10.1021/bi201075b PMC3172371

[68] Marincola G, Wolz C. Downstream element determines RNase Y cleavage of the saePQRS operon in *Staphylococcus aureus*. Nucleic Acids Res. 2017; 45:5980–94. 28453818 10.1093/nar/gkx296 PMC5449607

[69] Ludwig H, Homuth G, Schmalisch M et al. Transcription of glycolytic genes and operons in *B**acillus subtilis*: evidence for the presence of multiple levels of control of the gapA operon. Mol Microbiol. 2001; 41:409–22. 11489127 10.1046/j.1365-2958.2001.02523.x

[70] Meinken C, Blencke HM, Ludwig H et al. Expression of the glycolytic gapA operon in *Bacillus subtilis*: differential syntheses of proteins encoded by the operon. Microbiology. 2003; 149:751–61. 12634343 10.1099/mic.0.26078-0

[71] Le Scornet A, Jousselin A, Baumas K et al. Critical factors for precise and efficient RNA cleavage by RNase Y in *Staphylococcus aureus*. PLoS Genet. 2024; 20:e1011349. 39088561 10.1371/journal.pgen.1011349 PMC11321564

[72] McDowall KJ, Lin-Chaol S, Cohen SN. A+U content rather than a particular nucleotide order determines the specificity of RNase E cleavage. J Biol Chem. 1994; 269:10790–96. 7511606

[73] Lin-Chao S, Wong T-T, Mcdowall KJ et al. Effects of nucleotide sequence on the specificity of rne-dependent and RNase E-mediated cleavages of RNA I encoded by the pBR322 Plasmid. J Biol Chem. 1994; 269:10797–803. 7511607

[74] Yarchuk O, Jacques N, Guillerez J et al. Interdependence of translation, transcription and mRNA degradation in the lacZ gene. J Mol Biol. 1992; 226:581–96. 1507217 10.1016/0022-2836(92)90617-s

[75] Condon C. RNA processing and degradation in *Bacillus subtilis*. Microbiol Mol Biol Rev. 2003; 67:157–74. 12794188 10.1128/MMBR.67.2.157-174.2003 PMC156466

[76] Lehnik-Habrink M, Newman J, Rothe FM et al. RNase Y in *Bacillus subtilis*: a natively disordered protein that is the functional equivalent of RNase E from *Escherichia coli*. J Bacteriol. 2011; 193:5431–41. 21803996 10.1128/JB.05500-11 PMC3187381

[77] Hamouche L, Billaudeau C, Rocca A et al. Dynamic membrane localization of RNase Y in *Bac**illus subtilis*. mBio. 2020; 11:e03337–19. 32071272 10.1128/mBio.03337-19 PMC7029143

[78] Marincola G, Schäfer T, Behler J et al. RNase Y of *Staphylococcus**aureus* and its role in the activation of virulence genes. Mol Microbiol. 2012; 85:817–32. 22780584 10.1111/j.1365-2958.2012.08144.x

