# Supplementary material for: A high-resolution view of RNA endonuclease cleavage in Bacillus subtilis
Source: Nucleic Acids Res. 2025 Jan 30;53(3):gkaf030. doi: 10.1093/nar/gkaf030 (PMC11780869; doi:10.1093/nar/gkaf030)
Supplement: gkaf030_Supplemental_Files [file gkaf030_supplemental_files.zip › TaggartSupplemental_241231.pdf]

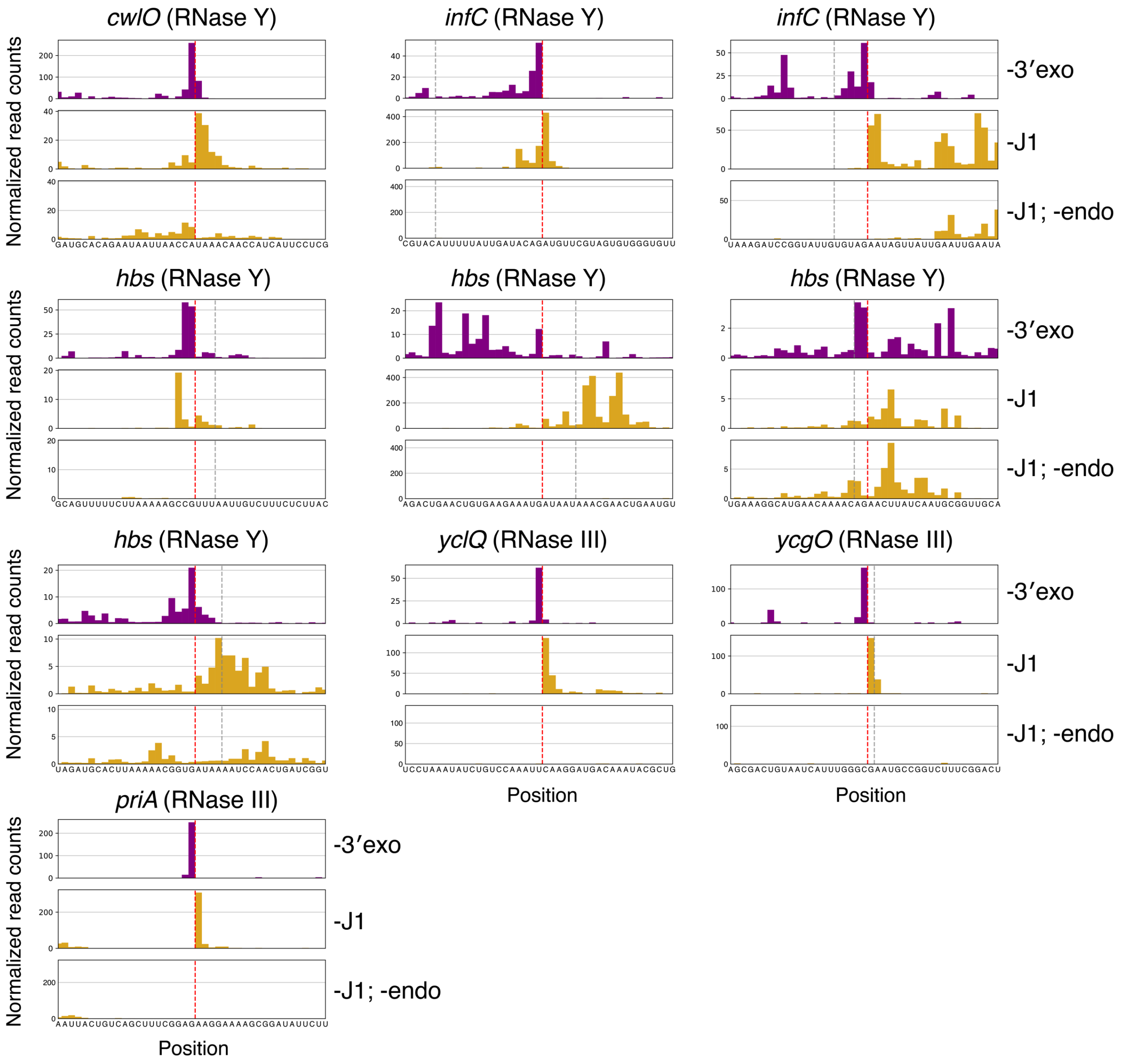

Figure S1. Validation of approach through detection of known positions of endonuclease activity. 5′ and 3′ end sequencing data at known positions of cleavage by RNases Y and III. Datasets considered are a 4-exo knockout (CCB396), *rnjA* knockout (CCB434), and either deletion or depletion of RNase J1 with a knockout of RNase Y (CCB760) or III (BG879). 5′-mapped data shown in yellow and 3′-mapped data shown in purple. Plotted are reads per million CDS-mapping reads, normalized to the average 3′-mapped Rend-seq RPM in this window. A manually annotated position of cleavage based on sequencing data is shown with a red dotted line, and the published position is marked in gray of cleavage if this position disagrees with our annotation. In transcripts with many nearby peak pairs such as *hbs*, additional cleavage positions may be visible beyond that which is highlighted.

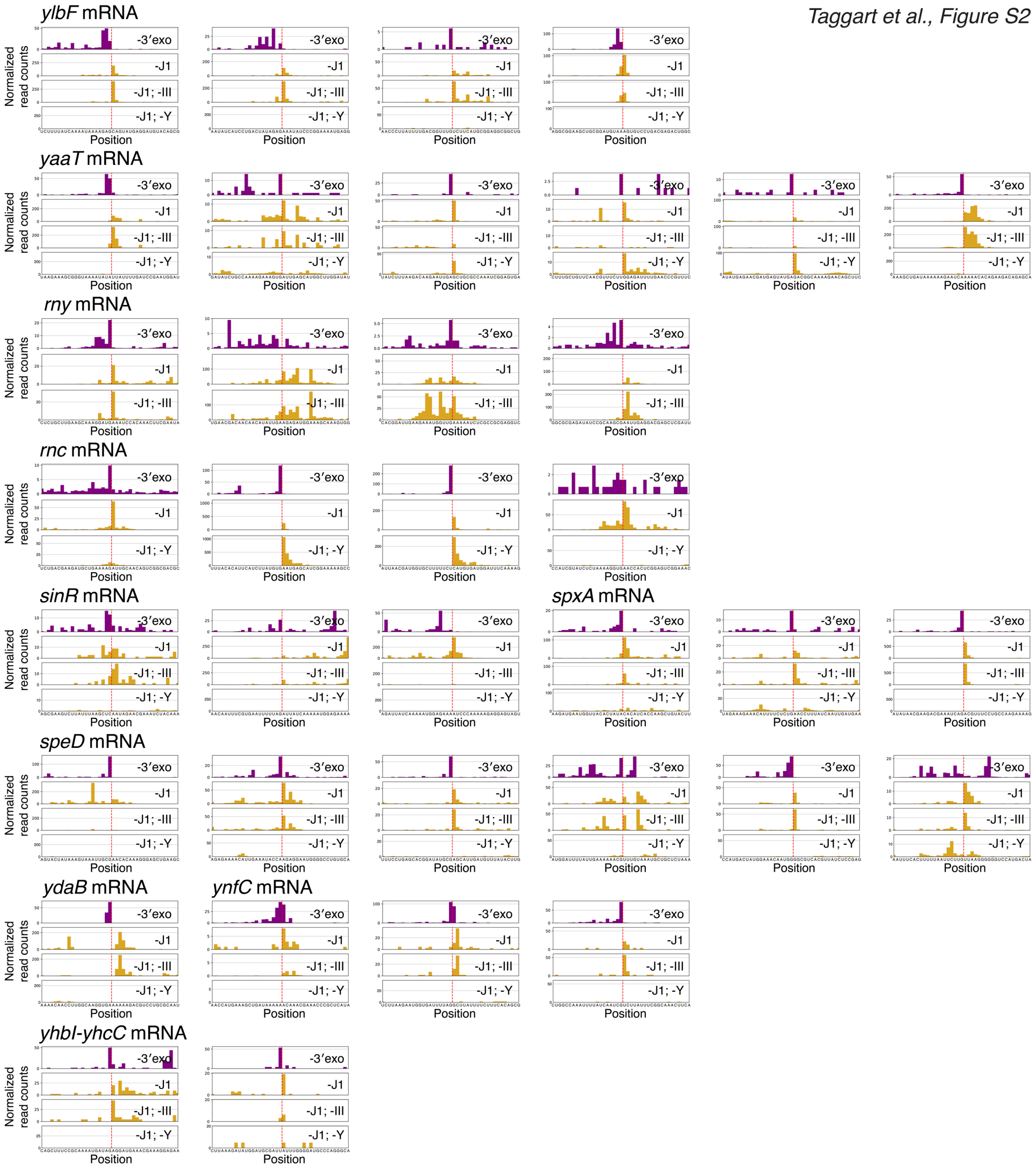

Figure S2. **Sequence context and RNase Y/III dependence of cleavage positions within mRNAs known to be destabilized by endoribonucleases**. Cleavage positions match order of those shown in Figure 2. Datasets considered are a 4-exo knockout (CCB396), *rnjA* knockout (CCB434), *rnjA rnc* double knockout in an SPβ and Skin-cured background (BG879), and depletion of RNase J1 with a knockout of *rny* (CCB760). 5′ end sequencing data shown in yellow and 3′ end sequencing data shown in purple. Plotted are reads per million CDS-mapping reads, normalized to the average 3′-mapped Rend-seq RPM in this window. A manually annotated

position of cleavage based on sequencing data is shown with a red dotted line. Data from the *rnc*- or *rny*-deficient strain are not shown for sites within the transcripts encoding these genes.

Taggart et al., Figure S3

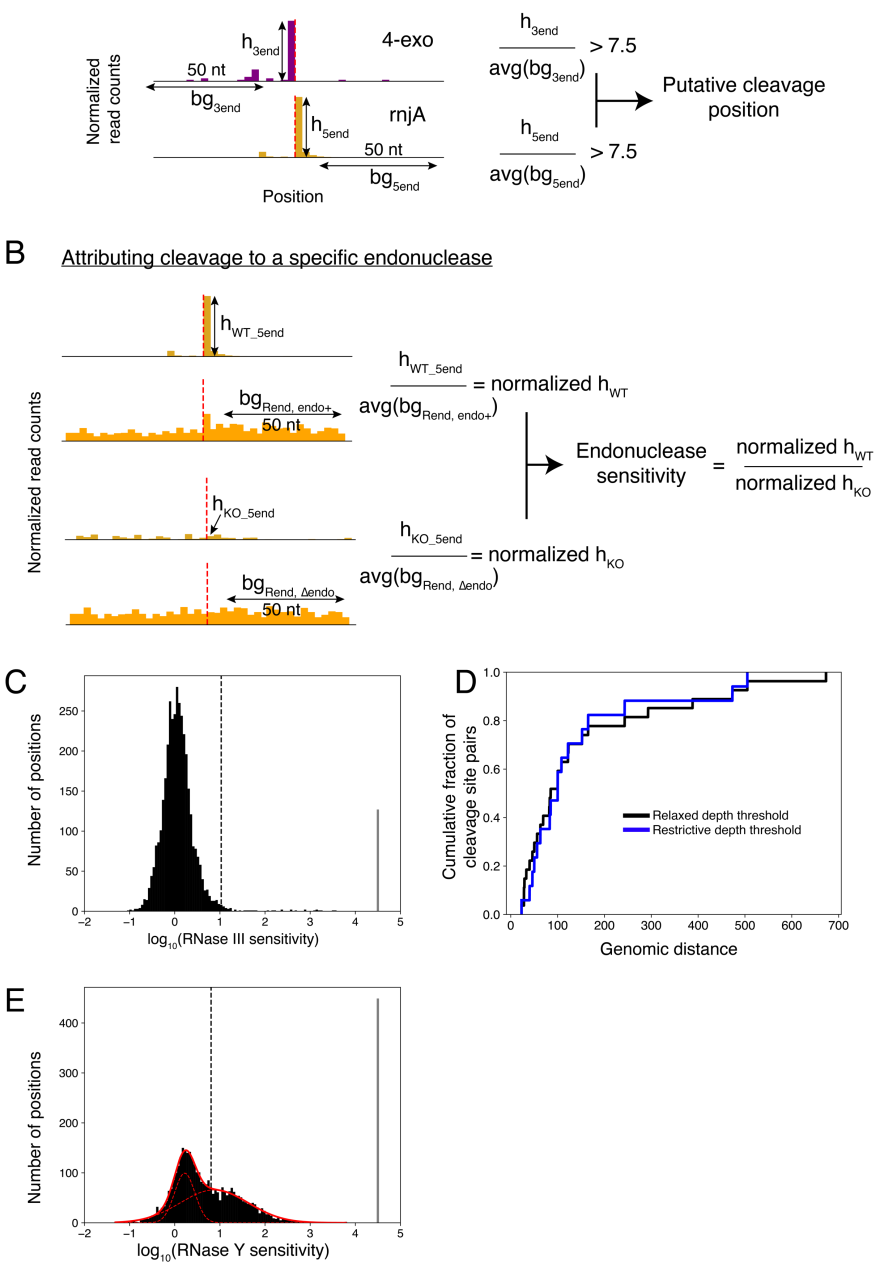

Figure S3. Schematic representation of strategy for calling putative cleavage positions and assigning them to the activity of endoribonucleases

(A) Strategy for calling paired 3′ and 5′ peaks in end sequencing data, with sites exceeding a peak-to-background ratio of 7.5 in the 3′/5′ at adjacent positions called as putative cleavage positions.

(B) Strategy for attributing sites to specific endonucleases. Peak heights in 5′ end sequencing are normalized to local density in a corresponding 5′-mapped Rend-seq dataset. A ratio of normalized peak heights between exoribonuclease strains with and without an endoribonuclease knockout (termed “endonuclease sensitivity”) is calculated as a metric for

dependence on this endoribonuclease. Note that prior to illustrated calculation, 5′ end sequencing data (h_{WT_5end} and h_{KO_5end}) are normalized by the sum of 5′ end sequencing reads mapped to putative cleavage positions, and the Rend-seq background (bg_{Rend}) is normalized to the total CDS-mapped reads to account for differences in sequencing depth between samples.
(C) Results of systematic identification of RNase III sites with a relaxed 5′ end sequencing depth threshold (as used in Figure 3G, H). Histogram shows distribution of endonuclease sensitivities for called peak pairs in 3′/5′ end sequencing of exoribonuclease knockouts (see Figure S3A and Methods), with black dashed line indicating threshold for calling dependence on RNase III. 174 sites exceeded this defined threshold. For 127 sites we were unable to calculate a sensitivity score due to an absence of 5′ end sequencing counts in our knockout. These sites are called as RNase III sensitive and are counted within the ”>4” bin of the histogram.
(D) Genomic distance between pairs of unambiguous RNase III cleavage positions identified within 1 kb of one another and predicted to fall on opposite sides of an RNA stem, using both restrictive and relaxed 5′ end sequencing depth thresholds.
(E) Results of systematic identification of RNase Y sites with a relaxed 5′ end sequencing depth threshold. Histogram shows distribution of RNase Y sensitivities, plotted as described in Figure 5A. 1477 sites exceeded this defined threshold. For 449 sites we were unable to calculate a sensitivity score due to an absence of 5′ end sequencing counts in our knockout. These sites are called as RNase Y sensitive and are counted within the ”>4” bin of the histogram.

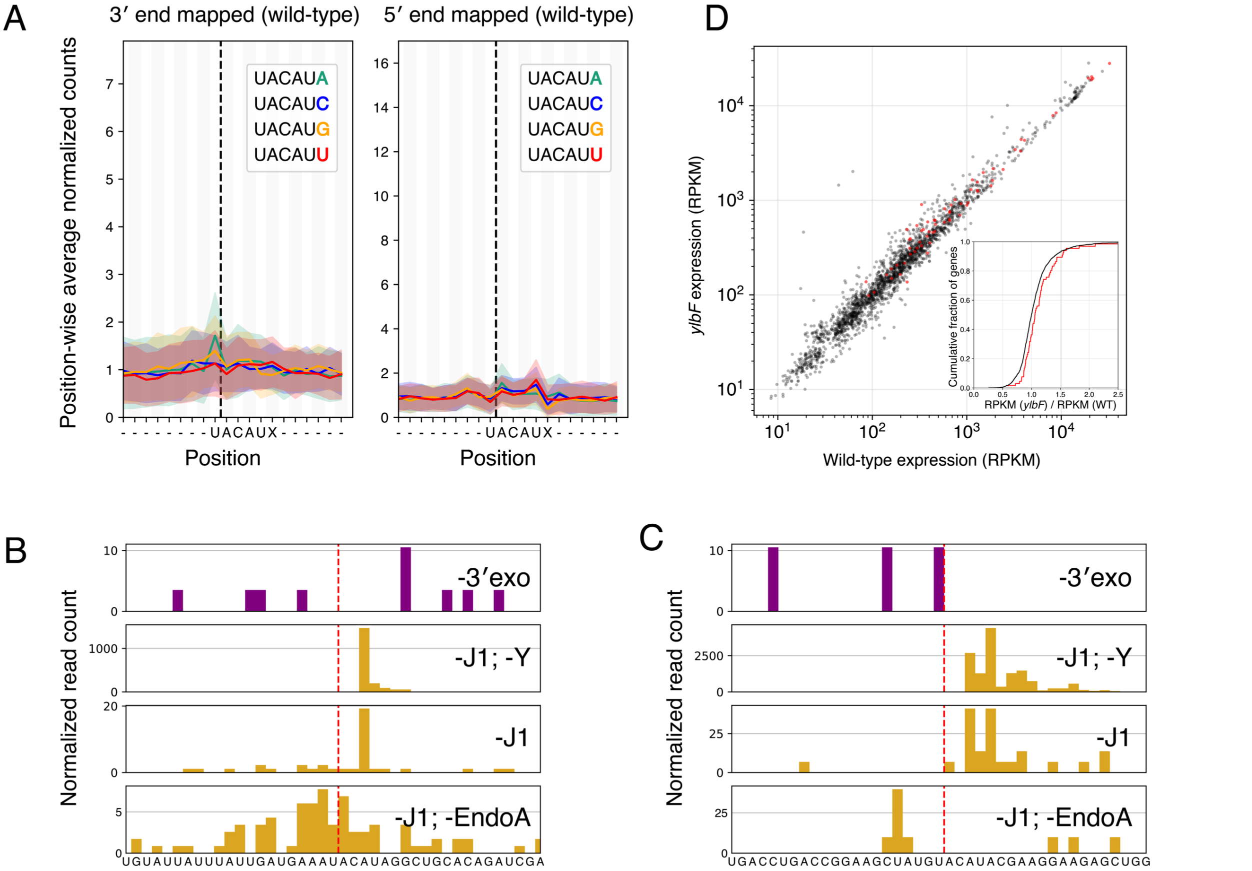

Figure S4. EndoA and YlbF have a limited influence on wild-type *B. subtilis* mRNA decay

(A) UACAUA-specific EndoA cleavage is not detectable in wild-type *B. subtilis.* 3′ (left) and 5′ (right) mapped Rend-seq signal across all UACAU motifs in the genome, separated by downstream nucleotide. Data are derived from wild-type W168. Rend-seq data at each site is normalized to first 8 (for 3′-mapped) or last 8 (for 5′-mapped) positions within the window and a position-wise mean and standard deviation are calculated with 90% winsorization. Motif instances with fewer than 1 read per position or fewer than 10 reads within the normalization window are not considered. Following this filtering, the number of considered sites ranges from 193 to 313 (for 3′-mapped) and 203 to 311 (for 5′-mapped) per motif. The dashed vertical line indicates the position of cleavage by EndoA.

(B, C) Representative EndoA sites showing absence (B) or presence (C) of subsequent trimming after the first two nucleotides. Yellow indicates 5′ end sequencing data and purple indicates 3′ end sequencing data. Plotted are reads per million CDS-mapping reads, normalized to the average 3′-mapped Rend-seq RPM in this window. Red dotted line represents manually annotated EndoA cleavage positions. Note that cleavage product accumulation is dependent on EndoA (bottom two panels).

(D) mRNA abundances for all annotated *B. subtilis* genes in a wild-type and *ylbF* W168 background. Lowly expressed genes (<128 total reads) are excluded. RPKM calculated as reads per thousand bases of the gene per million total reads that map to *B. subtilis* coding regions.

Inset shows differential expression distribution (Δ*ylbF*/WT RPKM) for genes with genes with (red) or without (black) a YlbF-dependent putative cleavage site.

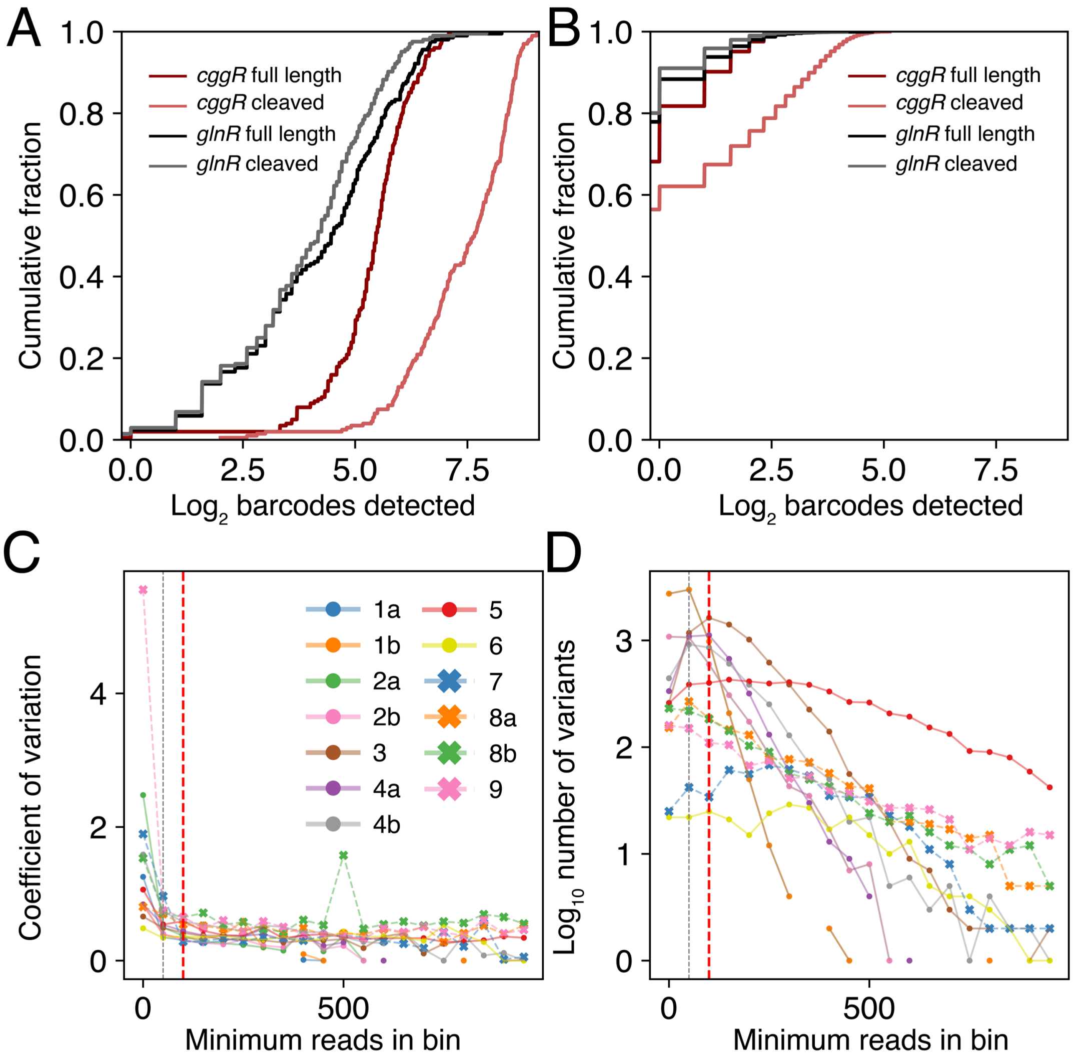

Figure S5. Variability and sequence coverage within our MPRA datasets

(A) Cumulative distribution of the number of unique barcodes associated with each possible sequence containing a single substitution relative to the wild-type *cggR* or *glnR* operon sequence. Undetected sequences are included with a pseudocount of 0.1 prior to log_2 transformation.

(B) Cumulative distribution of the number of unique barcodes associated with each possible sequence containing two substitutions relative to the wild-type *cggR* or *glnR* operon sequence. Undetected sequences are included with a pseudocount of 0.1 prior to log_2 transformation.

(C) Variation in MRPA readout as a function of genomic DNA sequencing depth. The normalized MPRA RNA abundance for each variant with a wild-type sequence was calculated as illustrated in Figure 6A. These values were grouped by the number of unique reads mapping to that variant in the genomic DNA barcode sequencing with a bin width of 50 reads. The coefficient of variation within each group is shown. The gray vertical dashed line indicates the 50 read cutoff

used for experiments 4b, 8a, 8b, and 9, red vertical dashed line indicates the 100 read cutoff used in analysis of all remaining experiments. See Table S7 for more information.

(D) The number of variants within each bin for each dataset show in (A). Dashed lines show thresholds as described in (A).

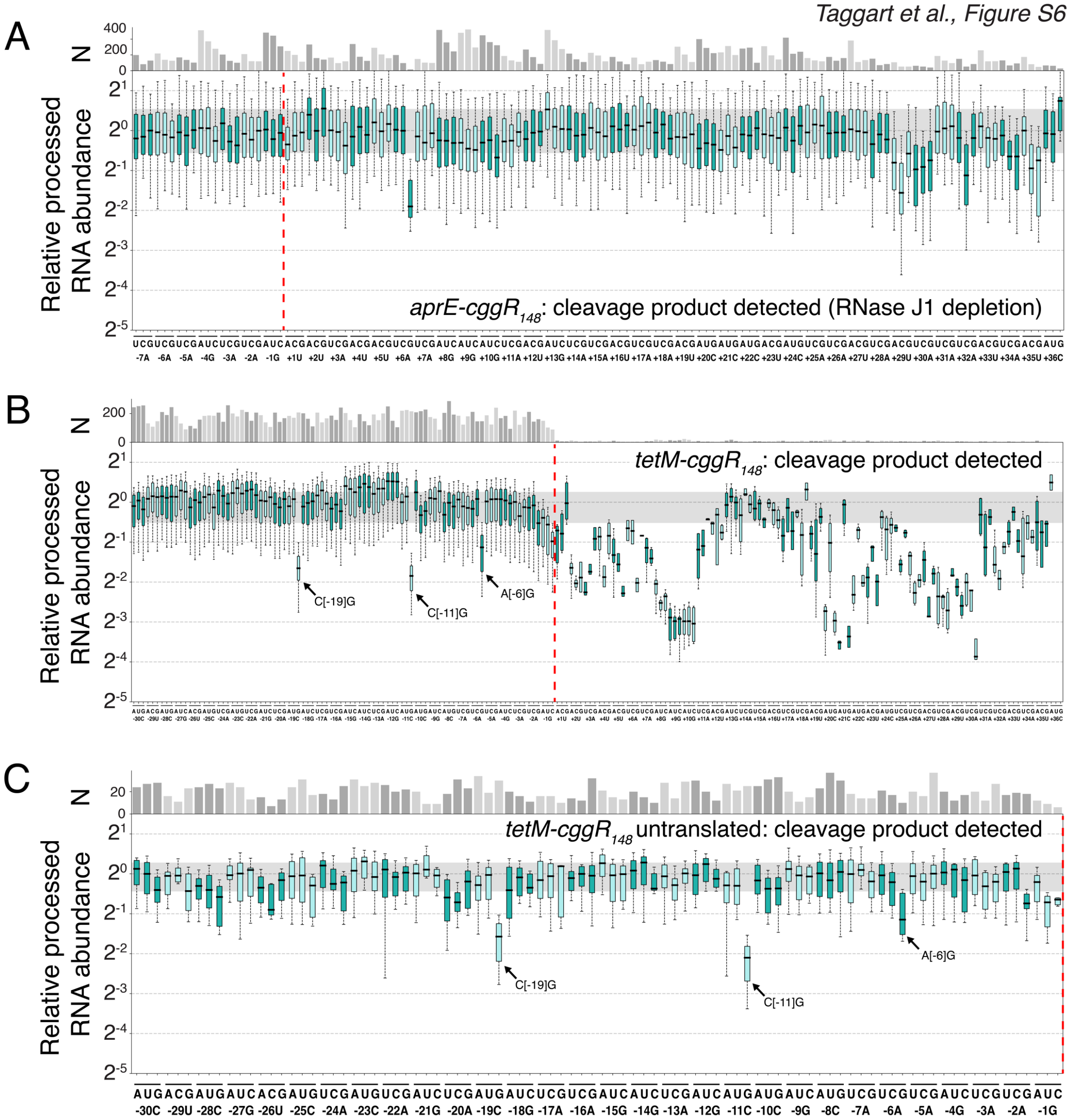

Figure S6. Features driving RNase Y cleavage within *cggR* are consistent across multiple MPRA contexts

(A) Impact of all single-nucleotide mutations on the accumulation of barcoded cleaved *aprE-cggR*$_{148}$ RNA in the context of RNase J1 depletion. Boxplots show variation between barcodes of identical variant sequence. Number of barcodes captured for each mutation is indicated above plot. Whiskers indicate 5th and 95th percentile. Gray shaded region indicates interquartile range for variants of wild-type sequence. Red line indicates position of cleavage. Ten variants have a value of zero (no more than one per mutation) and are thus not visualized.

(B) Impact of all single-nucleotide mutations on the accumulation of barcoded cleavage product when the *cggR₁₄₈* construct is appended to a new scaffold RNA, *tetM*. Plotted as in (A). Two variants (no more than one per mutation) have a value of zero are thus not visualized.
(C) Impact of all single-nucleotide mutations in the 30 nucleotides upstream of the $cggR_{148}$ cleavage site on the accumulation of barcoded cleavage product when a stop codon is inserted upstream of the inserted sequence. *tetM* scaffold was used in this experiment, as in (C). Plotted as in (A).

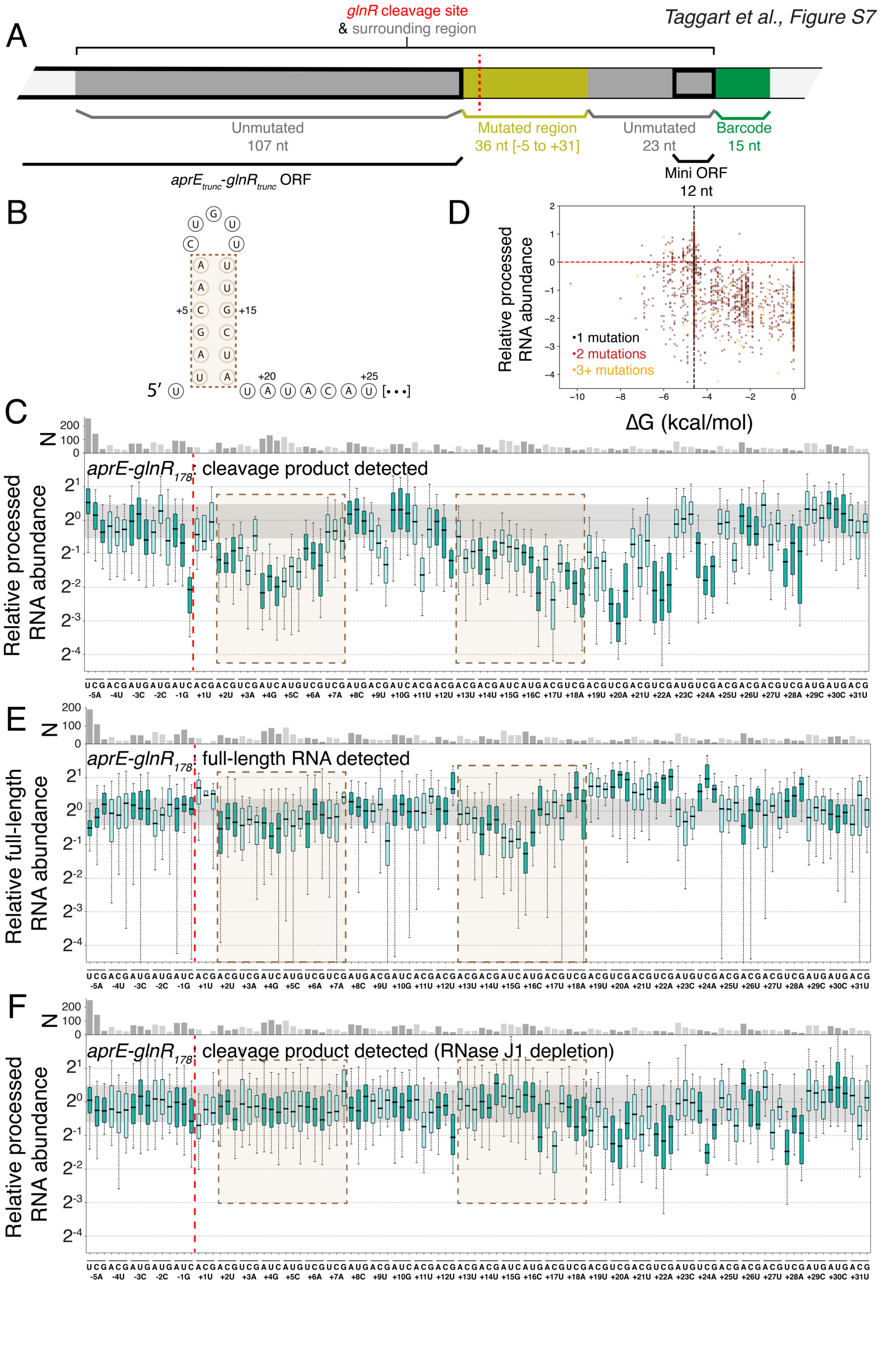

Figure S7. Determinants of *glnR-glnA* mRNA processing by RNase Y.

(A) Schematic of the *glnR*$_{178}$ construct inserted into the *aprE* MPRA transcript. *glnR*-derived sequence is colored dark gray, with the mutated positions highlighted in yellow. Translated regions are indicated with a thick border, and the variant barcode is indicated in green.
(B) Predicted stem-loop structure at 5′ end of processed RNA. Brown bar corresponds to regions highlighted in panels C, E, and F.
(C) Impact of all single-nucleotide mutations on the accumulation of barcoded cleaved RNA. Boxplots show variation between barcodes of identical variant sequence. Number of barcodes captured for each mutation is indicated above plot. Whiskers indicate 5th and 95th percentile. Gray shaded region indicates interquartile range for variants of wild-type sequence. Red line indicates position of cleavage. Brown bar corresponds to positions predicted to form the stem of the structure showed in (B). Nine variants (no more than two per mutation) have a value of zero are thus not visualized.
(D) Relationship between predicted strength of downstream secondary structure and accumulation of barcoded cleavage product. All data are derived from experiment 8a (Table S7). The vertical dashed line indicates the ΔG of the unmutated sequence. 17 variants fall outside of the bounds of this plot (Table S10).
(E) Impact of all single-nucleotide mutations on the accumulation of barcoded full-length RNA, plotted as described in (C). 30 variants (no more than two per mutation) have a value of zero are thus not visualized.
(F) Impact of all single-nucleotide mutations on the accumulation of barcoded cleaved RNA in the context of RNase J1 depletion, plotted as described in (C). Three variants (no more than one per mutation) have a value of zero are thus not visualized.

Table S1. List of strains used in this study

Table S2. List of oligonucleotide sequences used in this study

Table S3. List of identified RNase III cleavage sites

Table S4. List of identified RNase Y cleavage sites

Table S5. List of putative RNase III cleavage sites with relaxed 5' end sequencing depth threshold

Table S6. List of putative RNase Y cleavage sites with relaxed 5' end sequencing depth threshold

Table S7. Summary of MPRA experiments conducted in this study

Table S8. Data underlying Figure 6G

Table S9. Data underlying Figure 6H

Table S10. Data underlying Figure S7D
